# 2-APQC, a small-molecule activator of Sirtuin-3 (SIRT3), alleviates myocardial hypertrophy and fibrosis by regulating mitochondrial homeostasis

**DOI:** 10.1038/s41392-024-01816-1

**Published:** 2024-05-15

**Authors:** Fu Peng, Minru Liao, Wenke Jin, Wei Liu, Zixiang Li, Zhichao Fan, Ling Zou, Siwei Chen, Lingjuan Zhu, Qian Zhao, Gu Zhan, Liang Ouyang, Cheng Peng, Bo Han, Jin Zhang, Leilei Fu

**Affiliations:** 1 West China School of Pharmacy and Department of Biotherapy, Cancer Center and State Key Laboratory of Biotherapy, West China Hospital, Sichuan University, Chengdu, 610041 China; 2 Sichuan Engineering Research Center for Biomimetic Synthesis of Natural Drugs, School of Life Science and Engineering, Southwest Jiaotong University, Chengdu, 610031 China; 3 State Key Laboratory of Southwestern Chinese Medicine Resources, Hospital of Chengdu University of Traditional Chinese Medicine, School of Pharmacy, Chengdu University of Traditional Chinese Medicine, Chengdu, 611137 China; 4 School of Pharmaceutical Sciences, Health Science Center, Shenzhen University, Shenzhen, 518060 China; 5 School of Traditional Chinese Materia Medica, Key Laboratory of Structure-Based Drug Design & Discovery of Ministry of Education, Shenyang Pharmaceutical University, Shenyang, 110016 China

**Keywords:** Cardiology, Drug discovery, Cell biology, Computational biology and bioinformatics, Chemical biology

## Abstract

Sirtuin 3 (SIRT3) is well known as a conserved nicotinamide adenine dinucleotide^+^ (NAD^+^)-dependent deacetylase located in the mitochondria that may regulate oxidative stress, catabolism and ATP production. Accumulating evidence has recently revealed that SIRT3 plays its critical roles in cardiac fibrosis, myocardial fibrosis and even heart failure (HF), through its deacetylation modifications. Accordingly, discovery of SIRT3 activators and elucidating their underlying mechanisms of HF should be urgently needed. Herein, we identified a new small-molecule activator of SIRT3 (named 2-APQC) by the structure-based drug designing strategy. 2-APQC was shown to alleviate isoproterenol (ISO)-induced cardiac hypertrophy and myocardial fibrosis in vitro and in vivo rat models. Importantly, in SIRT3 knockout mice, 2-APQC could not relieve HF, suggesting that 2-APQC is dependent on SIRT3 for its protective role. Mechanically, 2-APQC was found to inhibit the mammalian target of rapamycin (mTOR)-p70 ribosomal protein S6 kinase (p70S6K), c-jun N-terminal kinase (JNK) and transforming growth factor-β (TGF-β)/ small mother against decapentaplegic 3 (Smad3) pathways to improve ISO-induced cardiac hypertrophy and myocardial fibrosis. Based upon RNA-seq analyses, we demonstrated that SIRT3-pyrroline-5-carboxylate reductase 1 (PYCR1) axis was closely assoiated with HF. By activating PYCR1, 2-APQC was shown to enhance mitochondrial proline metabolism, inhibited reactive oxygen species (ROS)-p38 mitogen activated protein kinase (p38MAPK) pathway and thereby protecting against ISO-induced mitochondrialoxidative damage. Moreover, activation of SIRT3 by 2-APQC could facilitate AMP-activated protein kinase (AMPK)-Parkin axis to inhibit ISO-induced necrosis. Together, our results demonstrate that 2-APQC is a targeted SIRT3 activator that alleviates myocardial hypertrophy and fibrosis by regulating mitochondrial homeostasis, which may provide a new clue on exploiting a promising drug candidate for the future HF therapeutics.

## Introduction

Heart failure (HF) is a complex clinical syndrome that develops with myocardial fibrosis, cardiac hypertrophy or other cardiovascular diseases and is characterized by abnormalities in the structure and function of the heart.^1^ Myocardial hypertrophy is a pathological compensatory response to cardiac dysfunction or injury that is closely associated with chronic HF.^2–4^ Additionally, myocardial hypertrophy is well known to play an important role in the progression of pathological cardiac remodeling, which can manifest as abnormal myocardial fibrosis.^5–7^ The pathological mechanisms of HF are complicated and remain to be elucidated. In fact, cardiac dysfunction is often accompanied by abnormal mitochondrial oxidation metabolism and energy metabolism.^8–11^ Therefore, the regulation of mitochondrial metabolism-related proteins and enzymes to improve cardiac metabolism disorders is a promising therapeutic strategy for HF.^12–15^

Notably, Sirtuin 3 (SIRT3) is an important histone deacetylase in mitochondria and is highly involved in mitochondrial dynamics, ROS production and redox homeostasis.^16–20^ Emerging studies have shown that SIRT3 can protect cardiomyocytes from hypertrophy, relieve myocardial fibrosis and improve HF.^12,21–23^ SIRT3 can improve cardiac fibrosis and cardiac function by deacetylating glycogen synthase kinase 3 β at Lys15 and inhibiting TGF-β/ Smad3-mediated expression of fibrosis-related proteins.^24^ SIRT3 can also inhibit the differentiation of muscle fiber cells through the signal transducer and activator of transcription 3-NFATc2 signaling pathway to prevent cardiac fibrosis.^25^ Moreover, as an important epigenetic target, SIRT3 is involved in posttranslational protein modification and deacetylation of mitochondrial proteins to regulate cardiovascular diseases.^26–28^ SIRT3 can inhibit the production of ROS through deacetylation of forkhead box O3 (FOXO3) to activate manganese superoxide dismutase 2 (MnSOD2) and catalase.^12^

Due to the protective role of SIRT3 in the heart, activation of SIRT3 can be a potential therapeutic strategy to treat HF. A series of small-molecule compounds have been demonstrated to act as SIRT3 activators or upregulate the expression of SIRT3 in HF for potential therapeutic purposes. For example, honokiol, a pharmacological SIRT3 activator, can block or even reverse the myocardial hypertrophy response and inhibit the differentiation of cardiac fibroblasts into myofibroblasts.^29^ Licorice isoflavone A exerts an anti-hypertrophic effect by upregulating the expression of SIRT3.^30^ LCZ696 induces upregulation of MnSOD2 through a SIRT3-dependent pathway to reduce myocardial oxidative stress and apoptosis in pressure-loaded HF.^31^ In addition, by activating SIRT3, Oroxylin A can promote the SIRT3-mediated expression of the *superoxide dismutase 2 (SOD2)* gene by regulating the DNA binding activity of *FoxO3A* and increase the activity of SOD2 by deacetylation.^32^ However, the effect of these aforementioned compounds on SIRT3 activation is not selective.^33^ Thus, novel targeted SIRT3 activators still remain to be discovered for HF therapy.

Protein kinase B (Akt), is a silk/threonine protein kinase with a molecular weight of approximately 60 kDa, which is composed of Akt1, Akt2 and Akt3.^34^ The phosphorylation at Thr308 site increases the kinase activity of Akt, which induces the occurrence and progression of myocardial hypertrophy by regulating various intracellular proteins.^35^ One of the downstream regulatory pathways of Akt is the mTOR pathway.^36^ mTOR can induce phosphorylation of p70S6K, thereby recruiting eukaryotic elongation factor 4E and synthesizing proteins, which regulate myocardial hypertrophy and heart failure.^37^ Thus, the Akt/mTOR/p70S6K signaling pathway plays an important regulatory role in the occurrence of myocardial hypertrophy and heart failure. PYCR1 is a housekeeping enzyme that participates in the final step of proline biosynthesis by catalyzing P5C to proline.^38^ It locates in mitochondria, which mainly involved in the conversion of glutamate to proline in diseases, especially cancer.^39^ In addition, SIRT3 can increase enzymatic activity by directly binding and deacetylating PYCR1 at the K228 site,^40^ implying that the activation of SIRT3 may improve cardiac diseases by inhibiting PYCR1-mediated oxidative stress.

In our previous studies, we identified two potential activating pockets of SIRT3 near the NAD^+^-binding pocket (Pocket **L** and Pocket **U**). In this study, targeting Pocket **L**, we discovered an effective SIRT3 activator, 2-APQC, which binds to and activates SIRT3. This activation influences key signaling pathways, including the mTOR-p70S6K, JNK, and TGF-β/Smad3, effectively reducing ISO-induced myocardial hypertrophy and fibrosis rat heart failure in vitro and in vivo models, and confirmed the targeting of 2-APQC in *SIRT3* knockout mice. According to our RNA-Seq analyses, we confirmed that 2-APQC can protect cardiac cells from ISO-induced damage by activating the SIRT3-PYCR1 axis to inhibit mitochondrial oxidative stress and improve cardiac function by activating AMPK-Parkin signaling to inhibit ISO-induced necrosis. Taken together, these results demonstrate that 2-APQC is a novel targeted small-molecule SIRT3 activator that alleviates myocardial hypertrophy and fibrosis by regulating mitochondrial homeostasis, which would shed new light on discovery of a potential drug candidate for HF treatment in the future.

## Results

### Structure-based screening of potential targeted SIRT3 activators

**Fig. 1:**
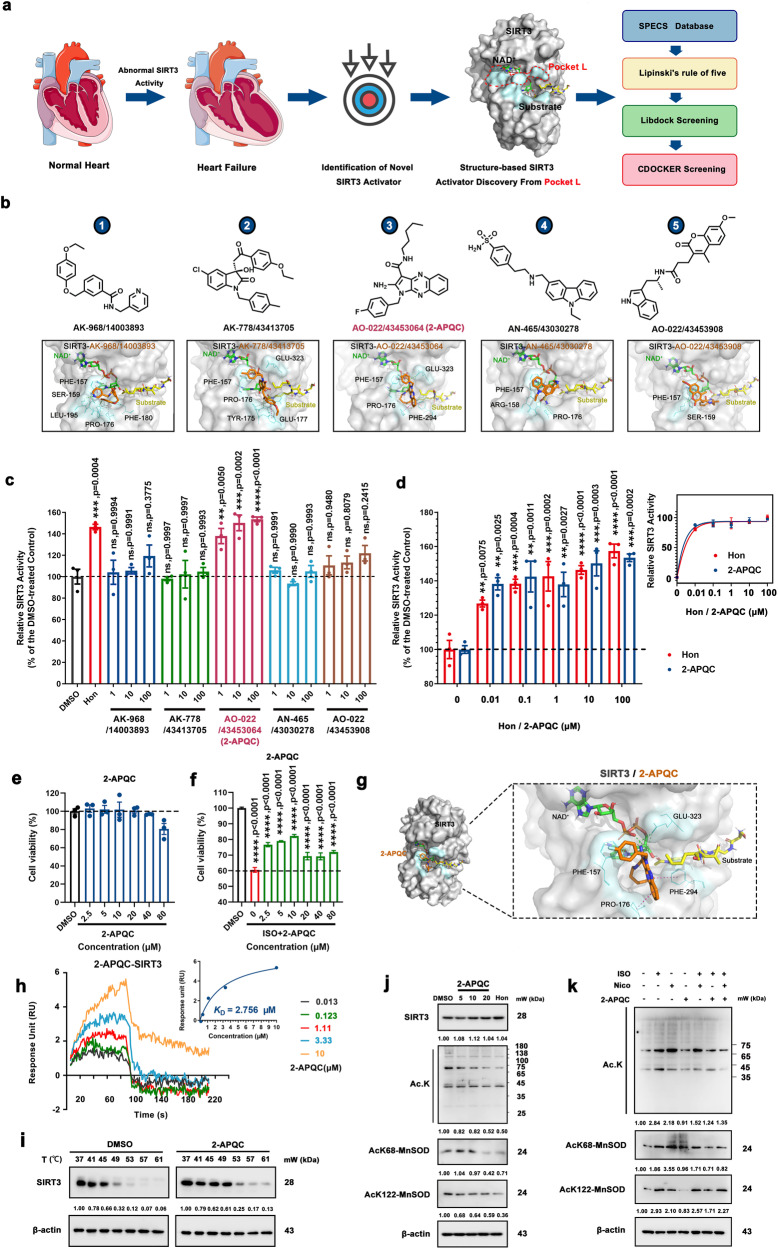
Structure-based screening of small-molecule activators of SIRT3 and identifies 2-APQC activates SIRT3 deacetylase activity in H9c2 cells. **a** The structure of SIRT3 and virtual screening scheme model for the discovery of SIRT3 activator. Created with biorender.com and https://smart.servier.com. **b** The 5 candidate compounds based on virtual screening and molecular docking. **c** The relative SIRT3 deacetylation activity after the candidate compounds (1, 10, 100 μM) and Honokiol (Hon) (10 μM) treatment. Data are present as mean ± s.e.m, *n* = 3. **d** The relative SIRT3 deacetylation activity after Hon or 2-APQC (0.01, 0.1 1, 10, 100 μM) treatment. Data are present as mean ± s.e.m, *n* = 3. **e** H9c2 cells were treated with indicated concentration of 2-APQC for 24 h, and cell viability was measured by the MTT assay. Data are present as mean ± s.e.m, *n* = 3. **f** H9c2 cells were treated with indicated concentration of 2-APQC for 24 h, and then treated with isoproterenol (ISO) for 48 h, the cell viability was measured by the MTT assay. Data are present as mean ± s.e.m, *n* = 3. **g** The molecular docking sketch map to indicate the binding mode between SIRT3 and 2-APQC. **h** Surface plasmon resonance assay detected the Kd value of SIRT3 in H9c2 cells treated with 2-APQC. **i** Cellular thermal shift assay detected the thermal stability of SIRT3 in H9c2 cells treated with 2-APQC. **j** Western blot analysis of SIRT3, acetylated-lysine, acetylated manganese superoxide dismutase 2 (MnSOD2) at K68 and K122 protein expression in H9c2 cells treated with 2-APQC for 24 h. **k** Western blot analysis of acetylated-lysine, acetylated MnSOD2 at K68 and K122 protein expression in H9c2 cells treated with 2-APQC for 24 h, together with or without ISO/nicotinamide (Nico) as the research design. β-actin, loading control. ns no significance; *p < 0.05, **p < 0.01, ***p < 0.001, ****p < 0.0001. Hon Honokiol, Nico nicotinamide, ISO isoproterenol

In this study, based on pocket **L**, we established a virtual screening strategy to identify potential small molecule SIRT3 activators (Fig. 1a). First, we obtained the top 20 potential compounds (Supplementary Table 1) by the LibDock protocol and CDOCKER protocol based on the SPECS database, and then we selected the top 5 candidates through structural diversity screening and molecular docking (Fig. 1b and Supplementary Fig. 2a). Next, we detected the relative SIRT3 deacetylation activity of these 5 candidates, of them Compound 3 (named 2-APQC) exhibited the greatest SIRT3 deacetylase activity (Fig. 1c). In addition, a dynamic simulation test also demonstrated the interaction between SIRT3 and 2-APQC (Supplementary Fig. 2b). Importantly, compared with another known SIRT3 activator, honokiol, 2-APQC showed comparable SIRT3 activation ability (Fig. 1d). We demonstrated that 2-APQC exhibited no apparent cytotoxicity even at higher concentrations (40 µM) (Fig. 1e), indicating that 2-APQC may have relatively good safety. In addition, 2-APQC could effectively ameliorate myocardial injury induced by ISO (Fig. 1f). The molecular docking results indicated that the binding pocket of 2-APQC highly overlaps with Pocket **L** of SIRT3. As the binding mode of 2-APQC with SIRT3 shows in Fig. 1g, 2-APQC could form hydrogen bonds with PHE157 and form hydrophobic interactions with residues PRO176, PHE294 and GLU323 (Fig. 1g). Surface plasmon resonance (SPR) also demonstrated a good interaction between 2-APQC and SIRT3, with an equilibrium dissociation constant Kd value of 2.756 μM (Fig. 1h). Thus, these results demonstrated that 2-APQC is a potential novel SIRT3 activator.

To prove that 2-APQC could directly bind and activate SIRT3 in cells, we first performed a cellular thermal shift assay (CETSA) in H9c2 cells. The results demonstrated that 2-APQC improved the thermal stability of SIRT3 in H9c2 cells, indicating that 2-APQC directly binds to SIRT3 (Fig. 1i). In addition, we tested the effects of different concentrations of 2-APQC treatment on the levels of SIRT1, SIRT2, SIRT4, SIRT5, SIRT6 and SIRT7, to investigate the selectivity of 2-APQC to SIRTs. The results showed that 2-APQC did not significantly affect the expression of SIRTs (Supplementary Fig. 3a). Through CETSA assay, it was demonstrated that 2-APQC did not improve the thermal stability of SIRT1, SIRT2, SIRT4, SIRT5, SIRT6 and SIRT7 in H9c2 cells, indicating that the binding stability of 2-APQC directly with SIRT1, SIRT2, SIRT4, SIRT5, SIRT6 and SIRT7 was poor (Supplementary Fig. 3b, c). Next, we tested whether the acetylation level of total lysine (Ac. k), acetylated MnSOD2 at lysine 68 (AcK68-MnSOD2) and AcK122-MnSOD2, which are known SIRT3 deacetylation sites of MnSOD, to check whether 2-APQC could enhance the deacetylation activity of SIRT3 in cells. The results showed that the levels of AcK68-MnSOD2 and AcK122-MnSOD2 were much lower in the presence of 2-APQC, and 2-APQC did not change the expression level of SIRT3 significantly (Fig. 1j). We also tested the effect of 2-APQC treatment on the level of SIRT3 protein and its substrates after different time periods and found 2-APQC changed the expression levels of AcK68-MnSOD2 and Acetylated lysine (Ac.K) proteins decreased with the extension of 2-APQC treatment time (Supplementary Fig. 3d). The results showed that 2-APQC time-dependently activated SIRT3 and enhanced its deacetylation activity. Furthermore, after treatment with the SIRT3 inhibitor nicotinamide (NICO), the deacetylation ability of SIRT3 substrates after 2-APQC treatment was significantly reduced, which also suggested the activation of cellular SIRT3 deacetylase activity after treatment with 2-APQC (Fig. 1k). Overall, these results indicated that 2-APQC could bind and activate SIRT3 deacetylation activity in vitro.

### 2-APQC improves HF by relieving myocardial hypertrophy and inhibiting myocardial fibrosis in ISO-induced HF model in vitro and in vivo

**Fig. 2:**
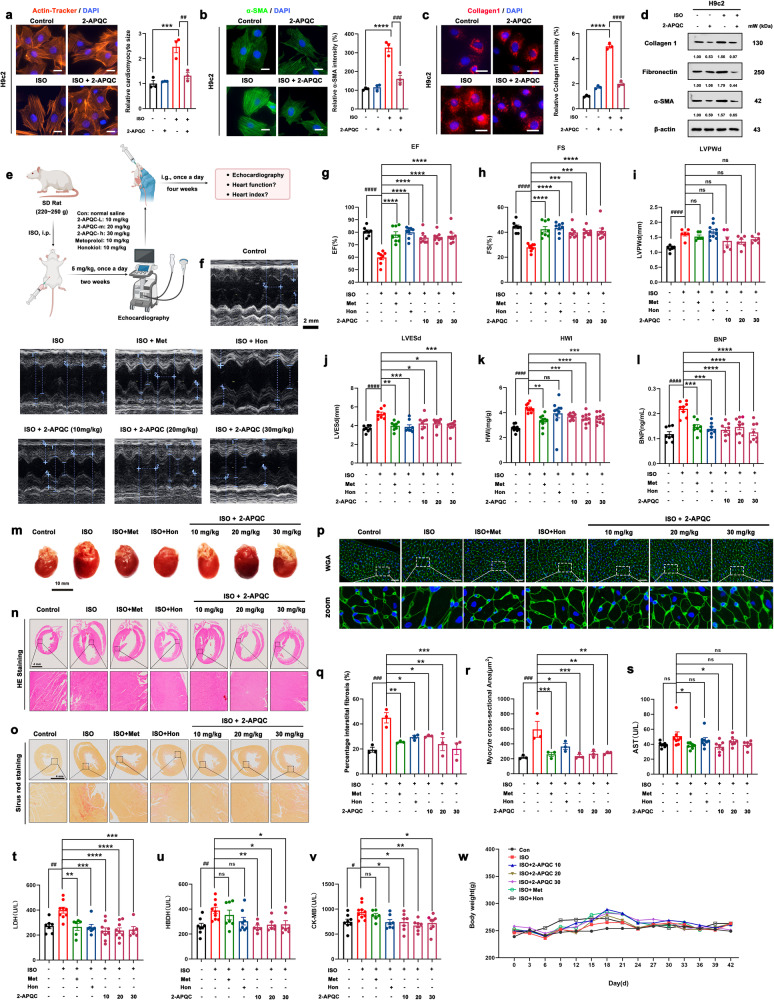
2-APQC improves heart failure (HF) by relieving myocardial hypertrophy and inhibiting myocardial fibrosis in ISO-induced HF model in vitro and in vivo. **a** H9c2 cells were treated with the indicated concentration 2-APQC for 24 h, and then ISO treated for 48 h. Determine the size of cardiomyocytes by β-actin tracker staining (red). The position of the nucleus is marked by 4’,6-diamidino-2-phenylindole (DAPI) staining. Scale bar = 10 μm. Data are present as mean ± s.e.m, *n* = 3. **b**, **c** Immunostaining of cells with α-SMA and Collagen. The position of the nucleus is marked by DAPI staining. The representative image is shown, and the Scale bar = 10 μm. Data are present as mean ± s.e.m, *n* = 3. **d** Detection and quantification of the expression of fibronectin and collagen I by Western blot. **e**, **f** Representative image of echocardiogram of indicated groups. **g**–**l** ejection fraction (EF), fractional shortening (FS), left ventricular posterior wall dimension (LVPWD) (mean ± s.e.m, con group, ISO group,ISO + Met group, ISO + 10 μM 2-APQC group, ISO + 20 μM 2-APQC group, ISO + 30 μM 2-APQC group, *n* = 6; ISO + Hon group, *n* = 10), LVESd, HWI (mean ± s.e.m, con group, *n* = 6; ISO group, ISO + Met group, ISO+Hon group, ISO + 10 μM 2-APQC group, ISO + 20 μM 2-APQC group, ISO + 30 μM 2-APQC group, *n* = 10), BNP (mean±s.e.m, con group, ISO group, ISO + Met group, ISO + Hon group, ISO + 10 μM 2-APQC group, ISO + 30 μM 2-APQC group, *n* = 8; ISO + 20 μM 2-APQ group, *n* = 10) were assessed by quantitative echocardiography, HWI was calculated by heart weight/body weight and Enzyme-linked immunosorbent assay was used to detect BNP levels. **m** Representative heart tissue in each group. **n** Represents the result of HE staining in heart tissues, scale bar = 4 mm. **o** Sirius Red staining in heart tissues, scale bar = 4 mm. *n* = 3. **p** Wheat germ agglutinin (WGA) staining in heart tissues, scale bar = 10 μm. **q** The percentage of fibrosis in heart tissue. The analysis of myocyte cross-sectional area in heart tissue. Data are present as mean ± s.e.m, *n* = 3. **r**–**v** Analysis of serum biochemical indicators. Use enzymatic reagent kit to measure serum AST (mean ± s.e.m, con group, ISO group, ISO + Met group, ISO + Hon group, ISO + 10 μM 2-APQC group, ISO + 20 μM 2-APQC group, *n* = 7; ISO + 30 μM 2-APQ group, *n* = 6), LDH (mean ± s.e.m, con group, ISO + Hon group, *n* = 7; ISO group, ISO + 10 μM 2-APQC group, ISO + 20 μM 2-APQC group, *n* = 9; ISO + Met group, ISO + 30 μM 2-APQ group, *n* = 6;), α-HBDH (mean ± s.e.m, con group, ISO + Hon group, *n* = 8; ISO group, *n* = 9; ISO + Met group, *n* = 7; ISO + 10 μM 2-APQC group, ISO + 20 μM 2-APQC group, *n* = 6; ISO + 30 μM 2-APQ group, *n* = 7;) and creatine kinase-MB (CK-MB) (mean ± s.e.m, con group, ISO + 30 μM 2-APQ group, *n* = 8; ISO group, *n* = 10; ISO + Met group, *n* = 6; ISO + Hon group, ISO + 10 μM 2-APQC group, ISO + 20 μM 2-APQC group, *n* = 7;). **w** The body weight changes during the experiment of the indicated groups. β-actin, loading control. ns, no significance, *p < 0.05, **p < 0.01, ***p < 0.001, ****p < 0.0001, compared with ISO group; ^#^*p* < 0.05, ^##^*p* < 0.01, ^###^*p* < 0.001, ^####^*p* < 0.0001, compared with control group. Nico nicotinamide, Hon Honokiol, ISO isoproterenol

Based on previous reports showing that SIRT3 protects cardiomyocytes from hypertrophic stimuli and fibrosis, we examined whether 2-APQC, as a SIRT3 activator, could also protect cardiomyocytes. A myocardial hypertrophy model and myocardial fibrosis model were established to further evaluate the protective effect of 2-APQC on cardiomyocytes. Cytoskeletal protein actin labeling with rhodamine-phalloidin was used to indicate cellular morphology. The immunofluorescence analysis results demonstrated that 2-APQC significantly alleviated the myocardial hypertrophy caused by ISO (Fig. 2a). α-smooth muscle actin (α-SMA) and collagen I are fibrosis-associated proteins,^41^ which were labeled with green and red fluorescence, respectively. The results demonstrated that ISO upregulated the expression of α-SMA and collagen I, while treatment with 2-APQC reduced the expression of these proteins (Fig. 2b, c). Further investigation of the expression levels of these proteins also confirmed that 2-APQC could reduce the ISO-induced increase in α-SMA, fibronectin and collagen I levels (Fig. 2d). Thus, 2-APQC can effectively protect myocardial cells from ISO injury to alleviate myocardial hypertrophy and myocardial fibrosis.

Since 2-APQC exhibits promising cardioprotective potential in vitro, we next established an ISO-induced rat HF model to investigate whether 2-APQC can improve myocardial injury in vivo, which is a more clinically relevant situation. The HF model was created by subcutaneous injection of 5 mg/kg ISO in rats for 2 weeks. Three different doses of 2-APQC were used: 10 mg/kg, 20 mg/kg and 30 mg/kg (Fig. 2e). First, the ISO-induced HF model was successfully established, and the drug efficacy was evaluated by echocardiography. β-receptor blockers can improve the survival rate of patients with chronic systolic HF (CHF).^42^ In this study, we used the β-receptor blocker metoprolol as a pharmacological positive control group in the treatment of HF. The results showed that 2-APQC, metoprolol and honokiol could improve heart function (Fig. 2f). The ejection fraction (EF), fractional short circuit (FS), left ventricular end systolic diameter (LVESD) and brain natriuretic peptide (BNP) are important indices that indicate cardiac systolic function.^43^ ISO injury decreased the EF (Fig. 2g) and FS (Fig. 2h) and increased the LVESD (Fig. 2j) and BNP (Fig. 2l), causing cardiac dysfunction, while 2-APQC and two positive control drugs improved ISO-induced cardiac systolic function. In addition, ISO increased the heart weight index (HWI) (Fig. 2k) and left ventricular posterior wall dimension (LVPWD) (Fig. 2i), which are characteristic of cardiac hypertrophy, and treatment with 2-APQC mitigated the increase in these indicators. In addition, by observing cardiac morphology, we found that ISO could induce substantial cardiac hypertrophy, which could be relieved by the positive control drugs and 2-APQC (Fig. 2m). The results of hematoxylin-eosin (HE) staining showed substantial myocardial injury and hypertrophy induced by ISO, and 2-APQC ameliorated the symptoms of ISO-induced cardiac dysfunction (Fig. 2n). Sirius Red is an acid red dye that can label collagen fibers by reacting with alkaline collagen fibers.^44^ We found that the collagen fiber abundance was significantly increased in the ISO group, and the abundance decreased after the addition of the positive control drugs and 2-APQC, indicating that 2-APQC could inhibit ISO-induced cardiac fibrosis (Fig. 2o). Wheat germ agglutinin (WGA) can specifically bind to a glycoprotein on the myocardial cell membrane and is used for observing cell morphology by labeling the cell membrane.^45^ Intriguingly, the results of WGA staining indicated that 2-APQC could reduce ISO-induced cardiac hypertrophy (Fig. 2p). The analysis of the percentage of interstitial fibrosis and the myocyte cross-sectional area also demonstrated that 2-APQC can improve ISO-induced cardiac fibrosis and hypertrophy (Fig. 2q, r). In addition, the serum levels of biochemical indicators lactate dehydrogenase (LDH), aspartate aminotransferase (AST), creatine kinase (CK)-MB and α-hydroxybutyrate dehydrogenase (α-HBDH) were also measured to evaluate myocardial injury (Fig. 2s-v). Myocardial injury results in the release of these proteins into the blood, causing the serum levels of these indicators to increase. Indeed, a significant increase in the levels of these indicators was found in the ISO group, while a decrease in the levels of these biomarkers was found in the 2-APQC, metoprolol and honokiol groups, indicating that 2-APQC can effectively protect rats from ISO-induced myocardial damage. In addition, the staining of HE organ tissue in the high-dose 2-APQC treatment group and the normal body weight of these groups during the experiment showed no obvious toxicity (Fig. 2w). The above experimental results indicate that 2-APQC plays a protective role in ISO-induced HF model rats by relieving myocardial hypertrophy and inhibiting myocardial fibrosis in vitro and in vivo.

### 2-APQC alleviates myocardial hypertrophy and fibrosis to improve HF by activating SIRT3 activity in vitro and in vivo

**Fig. 3:**
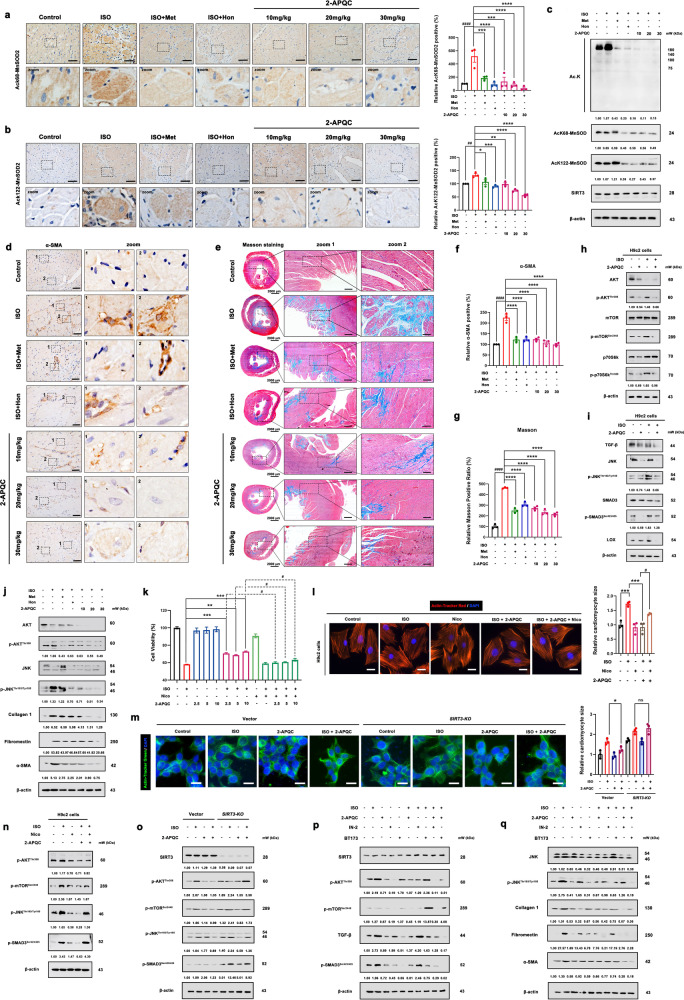
2-APQC alleviates myocardial fibrosis to improve HF by SIRT3 activation in vivo. **a**, **b** Immunohistochemical detection of ac-MnSOD2 (K68) and ac-MnSOD2 (K122) in rat heart tissues in different groups. Data are present as mean ± s.e.m, *n* = 3. **c** The expression levels of Ac-MnSOD2 (K68), ac-MnSOD2 (K122), SIRT3 and acetylated lysine were detected by Western blot. **d**, **f** Immunohistochemical detection of α-SMA cells with α-SMA in rat heart tissues in different groups. The position of the nucleus is marked by DAPI staining. Scale bar = 25 μm. Data are present as mean±s.e.m, n = 3. **e**, **g** Immunohistochemical detection of masson staining in rat heart tissues in different groups. Data are present as mean±s.e.m, n = 3. **h** Detection of Akt/ mTOR/p70S6K pathway by Western blot. **i** Detection and quantification of the expression of TGF-β/Smad3 pathway, TGF-β, JNK, p-JNK, Smad3, p-Smad3 and lysyl oxidase (Lox) by Western blot. **j** The expression levels of Akt, p-Akt, JNK, p-JNK, α-SMA, fibronectin and collagen I in rat heart tissues in different groups were detected by Western blot. **k** H9c2 cells were treated with indicated concentration of 2-APQC in the presence of absence of nicotinamide for 24 h, and then treated with ISO for 48 h, the cell viability was measured by the MTT assay. **l** H9c2 cells were treated with indicated concentration of 2-APQC in the presence of absence of nicotinamide for 24 h, and then treated with ISO for 48 h. Determine the size of cardiomyocytes by β-actin tracker staining (red). The position of the nucleus is marked by DAPI staining. Scale bar = 10 μm. Data are present as mean ± s.e.m, *n* = 3. **m** SIRT3 knockdown plasmid transfection was conducted 48 h prior to cell treatment. H9c2 cells were treated with indicated concentration of 2-APQC for 24 h, and then treated with ISO for 48 h. Determine the size of cardiomyocytes by β-actin tracker staining (green). The position of the nucleus is marked by DAPI staining. Scale bar = 10 μm. Data are present as mean ± s.e.m, *n* = 3. **n** Detect the expression of p-AKT, p-mTOR, p-JNK and p-SMAD3 by Western blot. **o** Detect the expression of p-AKT, p-mTOR, p-JNK and p-SMAD3 by Western blot in *SIRT3* KO mice. **p** Detect the expression of p-AKT, p-mTOR, TGF-β and p-SMAD3 by Western blot. **q** Detect the expression of JNK, p-JNK, α-SMA, fibronectin and collagen I by Western blot. ns, no significance, *p < 0.05, **p < 0.01, ***p < 0.001, ****p < 0.0001, compared with ISO group; ^#^*p* < 0.05, ^##^*p* < 0.01, ^###^*p* < 0.001, ^####^*p* < 0.0001, compared with control group. Nico nicotinamide, ISO isoproterenol

To check whether 2-APQC treatment of HF is related to the SIRT3 regulatory pathway, we examined the expression of ac-MnSOD2 (K68 and K122) by immunohistochemistry. The results showed that 2-APQC inhibited the acetylation of MnSOD2, which indicated the activation of SIRT3 (Fig. 3a, b). We extracted cardiac tissue and performed protein expression assays and found that 2-APQC reduced the expression level of acetylated lysine and MnSOD2 at K68 and K122 (Fig. 3c). In addition, the fibrosis protein marker α-SMA was significantly downregulated in the 2-APQC treatment group, indicating that 2-APQC can alleviate myocardial fibrosis (Fig. 3d, f). The Masson Staining results also showed that 2APQC could significantly alleviate ISO induced collagen fiber deposition phenomenon (Fig. 3e, g). Ribosomal kinase p70S6K, a downstream effector of the Akt/mTOR signaling pathway, has been reported to play an important role in myocardial hypertrophy.^46^ Herein, we checked key regulators of this pathway and found that the phosphorylation levels of AKT, mTOR and p70S6K were increased in the ISO group and decreased after treatment with 2-APQC, implying that 2-APQC could inactivate p70S6K via the Akt/mTOR signaling pathway to protect cardiomyocytes (Fig. 3h). Notably, TGF-β signaling mobilizes Smad2 and Smad3 transcription factors, which contribute greatly to fibrosis by promoting gene expression. The TGF-β-Smad3 pathway plays an important role in progressive cardiac fibrosis. TGF-β activates fibroblasts and promotes extracellular matrix production in diseased tissues, and its excessive deposition can mediate myocardial fibrosis.^47^ Here, we demonstrated that the phosphorylation of TGF-β, JNK, Smad3 and lysyl oxidase (Lox) in the 2-APQC + ISO group was lower than that in the ISO group (Fig. 3i). Compared with the control group, the treatment group exhibited decreased levels of phosphorylated AKT and JNK, and fiber associated proteins α-SMA, fibronectin, collagen I, further demonstrating that 2-APQC protects cardiomyocytes via the AKT/mTOR/p70S6K pathway and TGF-β/Smad3 pathway in rats. (Fig. 3j). Interestingly, the addition of nicotinamide, a SIRT3 inhibitor, attenuated the cardiomyocyte protective ability of 2-APQC, indicating that 2-APQC might protect cardiomyocytes from ISO damage by activating SIRT3 (Fig. 3k). Moreover, nicotinamide weakened the effect of 2-APQC on relieving myocardial hypertrophy, indicating that the treatment of myocardial hypertrophy by 2-APQC was dependent on SIRT3 activation (Fig. 3l). The fluorescence results of Actin staining showed that compared with the ISO group, the hypertrophy of myocardial cells in the ISO + 2-APQC group was reduced. After knocking out SIRT3, there was no significant change in myocardial cell hypertrophy symptoms in the ISO + 2-APQC group compared to the ISO group (Fig. 3m). In addition, the addition of the SIRT3 inhibitor nicotinamide increased the expression levels of p-Akt and p-mTOR, which were decreased by 2-APQC, suggesting that 2-APQC may inhibit the development of myocardial hypertrophy by activating the SIRT3-regulated Akt/mTOR signaling pathway (Fig. 3n). We also validated the pharmacological mechanism of 2-APQC in the ISO induced *SIRT3* knockout H9c2 heart failure cell model. The immunoblot results showed that compared with the ISO group, the expression levels of p-AKT, p-mTOR, p-JNK and p-Smad3 in the ISO + 2-APQC group were reduced. After knocking out *SIRT3*, compared with the ISO group, there was no significant change in the expression levels of p-AKT, p-mTOR, p-JNK and p-Smad3 in the ISO + 2-APQC group. Indicating that 2-APQC inhibits the mTOR/p70S6K pathway and TGF-β/Smad3 pathway by activating SIRT3 (Fig. 3o). To further validate the pharmacological mechanisms of 2-APQC in regulating the mTOR/p70S6K pathway and TGF-β/Smad3 pathway, we added PI3K/Akt/mTOR inhibitor IN-2 and TGF-β/Smad3 pathway inhibitor BT173 to the corresponding groups, respectively. After adding IN-2, the expression levels of p-AKT, p-mTOR, p-JNK, α-SMA, fibronectin, and collagen I decreased compared to the control group. Compared with the ISO + 2-APQC group, the expression levels of p-AKT, p-mTOR, p-JNK, α-SMA, fibronectin, and collagen I were reduced in the ISO + 2-APQC + IN-2 group (Fig. 3p, q). After BT173 treatment, the expression levels of TGF-β, p-Smad3, α-SMA, fibronectin, and collagen I in the BT173 group decreased compared to the control group. Compared with the ISO + 2-APQC group, the expression levels of TGF-β, p-Smad3, α-SMA, fibronectin, and collagen I were reduced in the ISO + 2-APQC + BT173 group (Fig. 3p, q), indicating that 2-APQC can inhibit myocardial cell fibrosis by regulating the mTOR/p70S6K pathway and TGF-β/Smad3 pathway. Moreover, after inhibiting mTOR signaling pathway and TGF-β/Smad3 signaling pathway respectively, the inhibitory effect of 2-APQC on myocardial hypertrophy and fibrosis marker was enhanced. These results demonstrated that 2-APQC can improve ISO-induced HF, myocardial hypertrophy and abnormal myocardial fibrosis by activating SIRT3 and suppressing the AKT/mTOR/p70S6K and TGF-β/Smad3 pathways.

### Effect of 2-APQC on ISO induced myocardial hypertrophy and fibrosis in *SIRT3* knockout mouse model

**Fig. 4:**
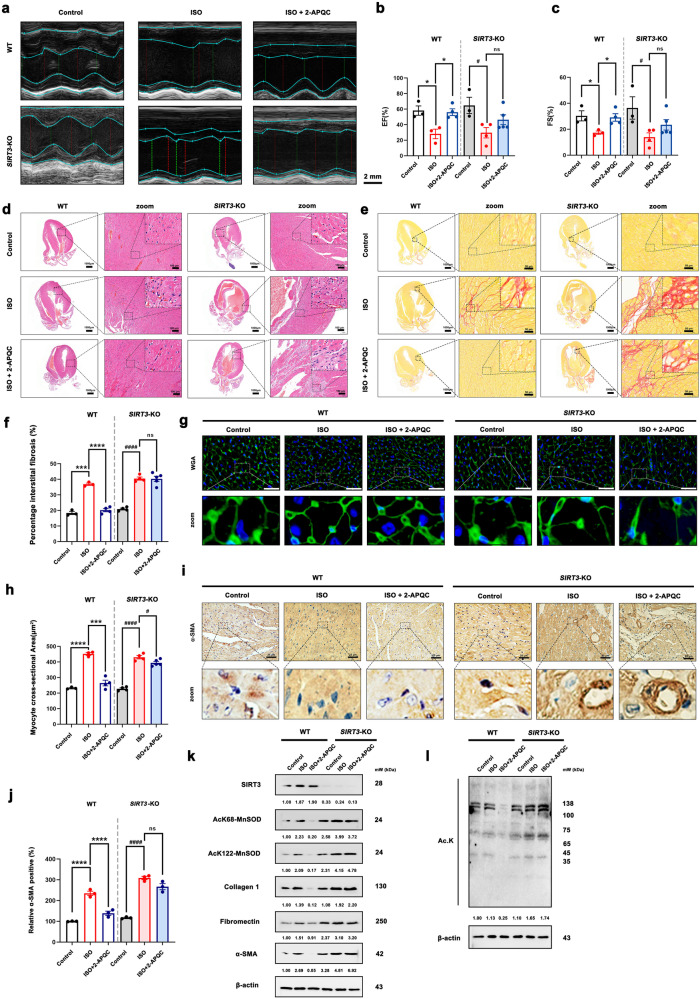
2-APQC could not alleviate ISO-induced HF in *SIRT3* knockout mouse model. **a**–**c** Representative image of echocardiogram of indicated groups. *n* = 3. **d** Represents the result of HE staining in heart tissues, scale bar = 1 mm; scale bar = 100 μm. **e**, **f** Sirius Red staining in heart tissues. Data are present as mean ± s.e.m, *n* = 3. **g**, **h** wheat germ agglutinin (WGA) staining in heart tissues, scale bar = 20 μm. Data are present as mean ± s.e.m, *n* = 3. **i**, **j** Represents the result of α-SMA staining in heart tissues, scale bar = 25 μm. Data are present as mean ± s.e.m, *n* = 3. **k**, **l** Detection and quantification of the expression of SIRT3, ac-MnSOD2 (K68), ac-MnSOD2 (K122) fibronectin, collagen 1 and α-SMA by Western blot. β-actin, loading control. ns, no significance, *p < 0.05, **p < 0.01, ***p < 0.001, ****p < 0.0001; ^#^*p* < 0.05, ^##^*p* < 0.01, ^###^*p* < 0.001, ^####^*p* < 0.0001. Nico nicotinamide, ISO isoproterenol, Hon Honokiol, Met metoprolol

We used echocardiography to evaluate the ejection fraction, fractional shortening and the myocardial protective effect of 2-APQC in vivo. Changes in cardiac function were analyzed through electrocardiogram imaging and quantitative analysis. Compared with the control group, the ISO group exhibited a decrease in EF% and FS%, indicating a decrease in cardiac systolic function. Compared with the ISO group, the 2-APQC treatment group showed increased EF% and FS% after four weeks of treatment. The ISO + 2-APQC group mice showed a decrease in EF% and FS% after *SIRT3* knockout, indicating that knocking out *SIRT3* further led to a decrease in EF% and FS%, eliminating the increase in EF% and FS% induced by 2-APQC (Fig. 4a–c). The results of the echocardiography analysis indicated that 2-APQC may improve ISO-induced cardiac dysfunction and exert cardioprotective effects by activating SIRT3 in ISO-induced heart failure mouse model. We used histopathology methods to investigate the effect of 2-APQC on the structure of cardiac tissue in HF mouse model. HE staining (Fig. 4d), Sirius red staining (Fig. 4e, f), WGA staining (Fig. 4g, h) and α-SMA staining (Fig. 4i, j) showed that compared with the control group, the ISO group and *SIRT3* knockout group exhibited substantial hypertrophy, significantly increased collagen levels in myocardial cells, abnormal collagen fibers around blood vessels and substantial fiber proliferation, and the area of α-SMA stained positive cells increased significantly. Compared with the ISO group, the ISO + 2-APQC group showed reduced myocardial hypertrophy, decreased collagen levels in muscle cells, decreased area of α-SMA staining positive cells, and alleviated fibroproliferation. After *SIRT3* knockout, myocardial hypertrophy worsened and the area of fibrosis increased in the ISO group. Compared with the ISO group, the ISO + 2-APQC group showed no improvement in symptoms of myocardial hypertrophy and fibrosis after *SIRT3* knockout. The above in vivo experimental results indicate that 2-APQC can improve systolic function, inhibit myocardial injury, and alleviate hypertrophy and fibrosis in an ISO-induced heart failure rat model by activating SIRT3. The western blotting results (Fig. 4k, l) also indicated that compared with the control group, the protein expression levels of fibronectin, collagen 1, α-SMA, and substrate ac-MnSOD2 (K68 and K122) in the ISO group and SIRT3 knockout group were significantly increased. Compared with the ISO group, the expression levels of fibrosis related proteins and ac-MnSOD2 (K68 and K122) were significantly reduced in the ISO + 2-APQC group. The expression levels of fibrosis related proteins and ac-MnSOD2 (K68 and K122) increased in the ISO group after SIRT3 knockout. After *SIRT3* knockout, the ISO + 2-APQC group showed no significant changes in the expression levels of fibrosis related proteins and ac-MnSOD2 (K68 and K122) compared to the ISO group, suggesting that 2-APQC could not improve HF when SIRT3 is deficiency. In summary, the above experimental results indicate that 2-APQC improves the systolic function of ISO induced in vitro and in vivo heart failure models by activating SIRT3.

### Transcriptomics-based identification of potential molecular mechanisms of HF improvement effect of 2-APQC in H9c2 cells

**Fig. 5:**
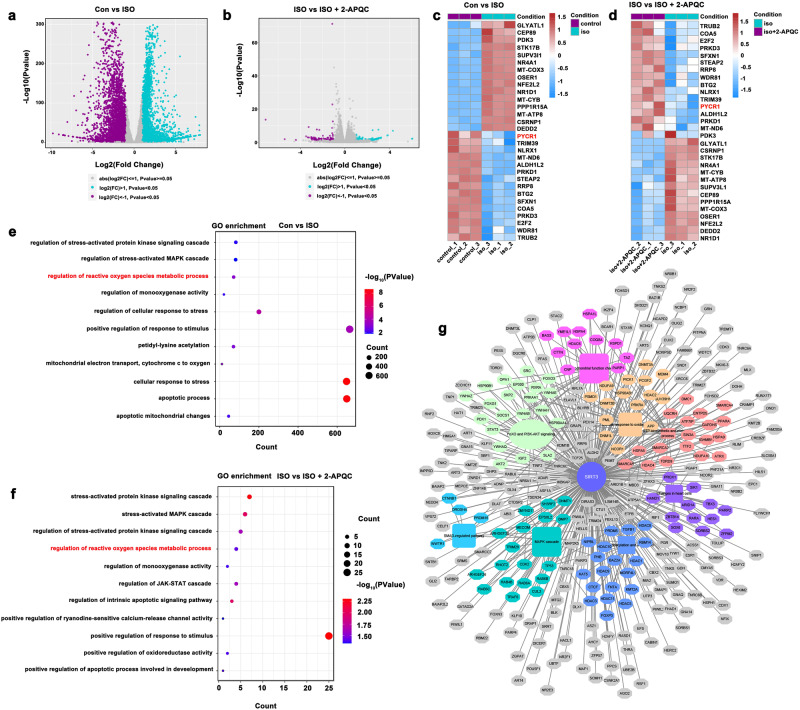
Transcriptomics-based identification of possible molecular mechanisms of HF improvement effect of 2-APQC in H9c2 cells. **a**, **b** The volcano plots showing the differential expression of genes in the heart tissues of CON VS ISO and ISO VS ISO + 2-APQC as shown. **c**, **d** Heat map analysis of genes differentially expressed in rat hearts of Con and ISO and ISO and ISO ISO + 2-APQC, grouping all related genes into hierarchical clusters. **e**, **f** GO analysis of genes differentially expressed in rat hearts of Con and ISO and ISO and ISO ISO + 2-APQC. **g** SIRT3-regulated PPI after 2-APQC treatment

To further clarify the potential mechanism of 2-APQC in HF, we treated H9c2 cells with 2-APQC for 24 h and ISO for 48 h and then performed RNA-seq analysis to check the differential expression of genes. RNA-seq results showed that there were 3790 upregulated and 3,531 downregulated differentially expressed genes between the control and ISO groups |log2(FC)|> 1, *P* value < 0.05) (Fig. 5a). There were 121 upregulated and 112 downregulated differentially expressed genes between the ISO and ISO + 2-APQC groups |log2(FC)|> 1, *P* value < 0.05) (Fig. 5b). Among these differentially expressed genes, we identified 30 genes involved in the biology of mitochondrial function, oxidative stress response and apoptosis (i.e., 15 upregulated and 15 downregulated genes). Among them, 2-APQC significantly upregulated the expression of *Wdr81*, *Steap2*, *Nlrx1* and *Pycr1*, and significantly downregulated the expression of *Nfe2l2* and *Oser1*, indicating that 2-APQC may protect cardiomyocytes by regulating these pathways (Fig. 5c, d, Supplementary Table 2). These differentially expressed genes were annotated using Gene Ontology (GO), which indicated that they are closely related to a variety of biological processes, including mitochondrial function and oxidative stress (Fig. 5e, f). In addition, we constructed a SIRT3-regulated PPI network and found that SIRT3 could improve cardiac function by modulating key regulators of a variety of biological processes (Fig. 5g). Overall, these results suggest that the regulation of oxidative stress may be the key to the anti-heart failure effect of 2-APQC.

### 2-APQC activates SIRT3-PYCR1-regulated proline generation to resist to mitochondrial oxidative stress and SIRT3-AMPK-receptor interacting protein kinase 3 (RIPK3)-modulated necrosis

**Fig. 6:**
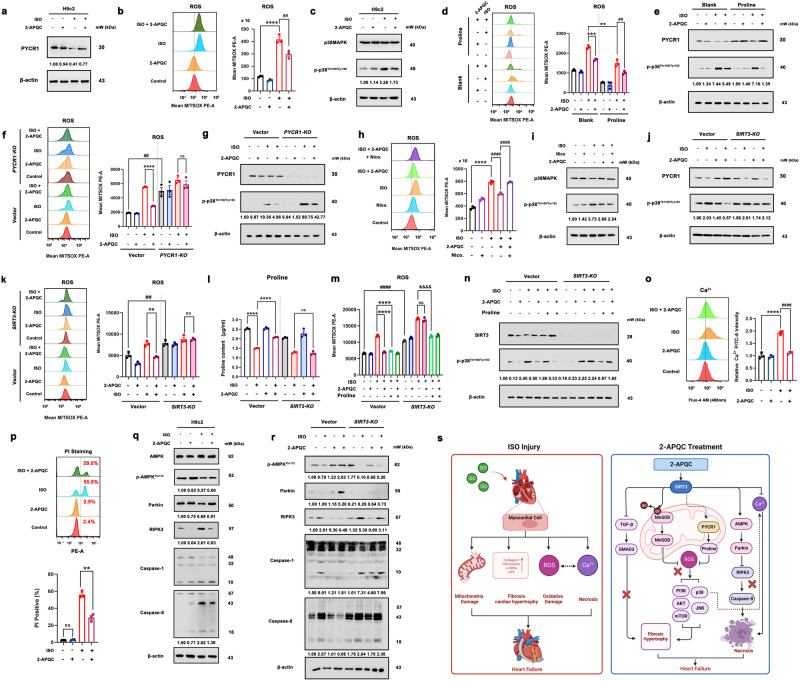
The SIRT3 activator 2-APQC plays a protective role in ISO-induced heart injury by SIRT3-pyrroline-5-carboxylate reductase 1 (PYCR1) regulated mitochondrial oxidative stress and SIRT3-AMPK-receptor interacting protein kinase 3 (RIPK3)-modulated necrosis. **a** H9c2 cells were treated with the specified concentration of 2-APQC for 24 h, and then treated with ISO for 48 h. The expression of PYCR1 was analyzed by western blot. β-actin was measured as a load control. The quantification of western blotting analysis is shown. **b** The content of reactive oxygen species (ROS) was measured by flow cytometry. The quantification of analysis is shown. Data are present as mean ± s.e.m, *n* = 3. **c** The expression of p38MAPK and p-p38MAPK was analyzed by western blotting. β-actin was measured as a load control. **d** H9c2 cells were treated with indicated concentration of 2-APQC in the presence of absence of proline for 24 h, and then treated with ISO for 48 h. The content of ROS was measured by flow cytometry. The quantification of analysis is shown. Data are present as mean ± s.e.m, *n* = 3. **e** H9c2 cells were treated with indicated concentration of 2-APQC in the presence of absence of proline for 24 h, and then treated with ISO for 48 h. The expression of PYCR1 and p-p38MAPK was analyzed by western blotting. β-actin was measured as a load control. **f** *PYCR1* knockdown plasmid transfection was conducted 48 h prior to cell treatment. H9c2 cells were treated with the specified concentration of 2-APQC for 24 h, and then treated with ISO for 48 h. The content of ROS was measured by flow cytometry. The quantification of analysis is shown. Data are present as mean ± s.e.m, *n* = 3. **g** *PYCR1* knockdown plasmid transfection was conducted 48 h prior to cell treatment. H9c2 cells were treated with the specified concentration of 2-APQC for 24 h, and then treated with ISO for 48 h. The expression of PYCR1 and p-p38MAPK was analyzed by western blotting. β-actin was measured as a load control. **h** The content of ROS was measured by flow cytometry. The quantification of analysis is shown. Data are present as mean ± s.e.m, *n* = 3. **i** H9c2 cells were treated with the specified concentration of 2-APQC and nicotinamide for 24 h, and then treated with ISO for 48 h, western blot analysis of p38MAPK and p-p38MAPK expressions. β-actin was measured as a load control. **j** *SIRT3* knockdown plasmid transfection was conducted 48 h prior to cell treatment. H9c2 cells were treated with the specified concentration of 2-APQC for 24 h, and then treated with ISO for 48 h. The expression of PYCR1 and p-p38MAPK was analyzed by western blotting. β-actin was measured as a load control. **k** *SIRT3* knockdown plasmid transfection was conducted 48 h prior to cell treatment. H9c2 cells were treated with the specified concentration of 2-APQC for 24 h, and then treated with ISO for 48 h. The content of ROS was measured by flow cytometry. The quantification of analysis is shown. Data are present as mean ± s.e.m, *n* = 3. **l** *SIRT3* knockdown plasmid transfection was conducted 48 h prior to cell treatment. H9c2 cells were treated with indicated concentration of 2-APQC in the presence of absence of proline for 24 h, and then treated with ISO for 48 h. Detect the proline content levels of each group, the quantification of analysis is shown. Data are present as mean ± s.e.m, n = 3. **m** *SIRT3* knockdown plasmid transfection was conducted 48 h prior to cell treatment. H9c2 cells were treated with the specified concentration of 2-APQC for 24 h, and then treated with ISO for 48 h. The content of ROS was measured by flow cytometry. The quantification of analysis is shown. Data are present as mean ± s.e.m, *n* = 3. **n** *SIRT3* knockdown plasmid transfection was conducted 48 h prior to cell treatment. H9c2 cells were treated with indicated concentration of 2-APQC in the presence of absence of proline for 24 h, and then treated with ISO for 48 h. The expression of SIRT3 and p-p38MAPK was analyzed by western blotting. β-actin was measured as a load control. **o** The content of Ca^2+^ was measured by flow cytometry. The quantification of analysis is shown. Data are present as mean ± s.e.m, *n* = 3. **p** H9c2 cells were treated with the specified concentration of 2-APQC for 24 h, and then treated with ISO for 48 h. Flow cytometry was used to detect the level of cell necrosis after PI staining. The quantification of analysis is shown. Data are present as mean ± s.e.m, *n* = 3. **q** The expression of AMPK, p-AMPK, Parkin, RIPK3, Caspase-1, Caspase-8 was analyzed by western blotting. **r** *SIRT3* knockdown plasmid transfection was conducted 48 h prior to cell treatment. H9c2 cells were treated with the specified concentration of 2-APQC for 24 h, and then treated with ISO for 48 h. The expression of p-AMPK, Parkin, RIPK3, Caspase 1, Caspase 8 was analyzed by western blotting. β-actin was measured as a load control. **s** 2-APQC, a small-molecule activator of SIRT3, alleviates heart failure by regulating SIRT3-mediated mitochondrial proline and ROS metabolic homeostasis. Created with biorender.com and https://smart.servier.com/. Data are expressed as mean ± SEM. All data represent at least three independent experiments. ns, no significance; *p < 0.05, **p < 0.01, ***p < 0.001, ****p < 0.0001, compared with control group; ^#^*p* < 0.05, ^##^*p* < 0.01, ^###^*p* < 0.001, ^####^*p* < 0.0001, compared with ISO group. ISO isoproterenol

Given that PYCR1 can enhance the biosynthesis of proline through deacetylation to combat oxidative stress.^40,48^ We measured the expression level of PYCR1 and found that 2-APQC reversed ISO-induced downregulation of PYCR1 (Fig. 6a). In addition, since ROS are one of the most important regulators of mitochondrial oxidative stress, we investigated the level of ROS, and the results showed that the ROS level in H9c2 cells increased after ISO treatment, and 2-APQC treatment significantly reduced this increase (Fig. 6b). In addition, compared with the ISO group, the addition of 2-APQC or antioxidant N-Acetyl-l-cysteine (NAC) improved the cell viability of H9c2 cells, suggesting that oxidative damage is indeed an important cause of ISO-induced cell viability reduction. However, 2-APQC cannot synergistically enhance the antioxidant capacity of NAC (Supplementary Fig. 4), indicating that the antioxidant activity of 2-APQC was comparable to that of NAC, and both were mainly achieved by scavenging ROS. Notably, it has been reported that p38MAPK controls the adaptive response of cells to stressful stimuli, and its function is closely related to cardiac development.^49^ Interestingly, p38MAPK can be activated by ROS stimulation. The results of this study showed that p-p38MAPK levels were significantly increased in the ISO group but decreased after 2-APQC treatment, while the total p38 levels did not vary significantly between groups (Fig. 6c). After supplementing with proline, we detected mitochondrial ROS levels and protein changes in p-p38MAPK. We found that after supplementing with proline, compared with the ISO group, the mitochondrial ROS levels in the ISO + proline group decreased, and the protein expression levels of p-p38MAPK decreased. Compared with the ISO + 2-APQC group, the ISO + 2-APQC+proline group showed a decrease in mitochondrial ROS levels and a decrease in protein expression levels of p-p38MAPK. It is suggested that supplementing with proline can reduce oxidative stress caused by ISO and synergistically enhance the protective effect of 2-APQC (Fig. 6d, e).

To further confirm the role of PYCR1 in 2-APQC-induced cell protection, we knocked out *PYCR1* and detected mitochondrial ROS levels and protein changes in p-p38MAPK, and found that after *PYCR1* knockout, compared with the negative control (NC)-ISO group, the mitochondrial ROS levels in the *PYCR1*-(knockout) KO + ISO group increased, and the protein expression levels of p-p38MAPK increased. Compared with the NC-ISO group+2-APQC group, the mitochondrial ROS level in the *PYCR1*-KO + ISO + 2-APQC group increased, and the protein expression levels of p-p38MAPK increased. Indicating that knocking out *PYCR1* can exacerbate the oxidative loss caused by ISO and weaken the protective effect of 2-APQC (Fig. 6f, g). The addition of the SIRT3 inhibitor NICO reversed the reduced ROS level of 2-APQC, illustrating that 2-APQC reduces ROS levels in myocardial cells by activating SIRT3 (Fig. 6h). In addition, nicotinamide treatment reversed the p-p38MAPK expression mediated by 2-APQC in ISO-induced H9c2 cells (Fig. 6i), indicating that 2-APQC may alleviate HF by inhibiting p38MAPK pathway. Then we knocked out *SIRT3*, and western blotting results showed that after knocking out *SIRT3*, there was no change in the expression levels of p-p38MAPK and PYCR1 in the ISO + 2-APQC group compared to the ISO group (Fig. 6j), indicating the loss of function of 2-APQC. Correspondingly, after knocking out *SIRT3*, there were no significant changes in mitochondrial ROS levels and proline content in the ISO + 2-APQC group compared to the ISO group (Fig. 6k, l). Indicating that 2-APQC inhibits oxidative stress by activating SIRT3 to regulate the PYCR1/p38MAPK axis. To verify the relationship between 2-APQC and SIRT3 and proline, after knocking out *SIRT3*, we supplemented proline in different groups and detected the protein changes of p-p38MAPK by detecting mitochondrial ROS levels. The results showed that after knocking out *SIRT3*, compared with the ISO group+2-APQC group, the mitochondrial ROS level in the ISO + 2-APQC+proline group decreased, and the protein expression level of p-p38MAPK decreased. After knocking out *SIRT3*, supplementing with proline can reduce ISO induced oxidative stress and partially enhance the protective effect of 2-APQC (Fig. 6m, n). These results suggest that PYCR1 is downstream of SIRT3, and the proline metabolism regulated by SIRT3-PYCR1 is very important in ROS clearance.

Considering that ROS are closely related to the regulation of cellular calcium ions, we next examined the effect of 2-APQC on the Ca^2+^ levels in H9c2 cells, which, when overloaded, can lead to cell necrosis and HF.^50^ The results showed that ISO can increase the level of Ca^2+^, while 2-APQC reduced the ISO-induced increase in Ca^2+^ levels, suggesting that 2-APQC may play a protective role in the heart by alleviating Ca^2+^ overload (Fig. 6o). Using flow cytometry, we demonstrated a decrease in the proportion of necrotic cells in the ISO + 2-APQC group compared to the ISO group (Fig. 6p). Intriguingly, p38MAPK is also involved in cardiomyocyte Ca^2+^ regulation, affecting cardiac contractile function. During cardiac contraction, the p38 pathway promotes the release of Ca^2+^ from the sarcoplasmic reticulum.^49^ Considering that the overload of Ca^2+^ can lead to cell death and that RIPK1, caspase 8 and Fas-associated protein (FADD) can interact to form the RIPK1-FADD-caspase 8 complex, causing pathological cardiac remodeling and HF by inducing necrotic cell death,^51,52^ we examined the effect of 2-APQC on this signaling pathway. The AMPK-Parkin-RIPK3 axis is a novel pathway that can negatively regulate necroptosis.^53^ AMPK can be activated by SIRT3,^54^ and we found that ISO can downregulate the expression of p-AMPK and Parkin, while treatment with 2-APQC can reverse this downregulation. Moreover, the expression levels of RIPK3 and caspase-8 were significantly increased in the ISO group but decreased after treatment with 2-APQC, suggesting that 2-APQC may improve cardiac function by activating the AMPK-Parkin-RIPK3 axis to inhibit necrosis (Fig. 6q). We also validated the pharmacological mechanism of 2-APQC in the ISO induced *SIRT3* knockout H9c2 heart failure cell model. The immunoblot results showed that compared with the ISO group, the expression levels of RIPK3, Caspase 1, and Caspase 8 in the ISO + 2-APQC group decreased, while the expression levels of p-AMPK and Parkin significantly increased. After knocking out *SIRT3*, there was no change in the expression levels of RIPK3, Caspase 1, Caspase 8, p-AMPK, and Parkin in the ISO + 2-APQC group compared to the ISO group, elucidating that 2-APQC regulates the AMPK-Parkin axis by activating SIRT3 to inhibit the formation of RIPK-FADD-Caspase 8 complex (Fig. 6r). The above experimental results illustrated that 2-APQC could protect cardiac cells from ISO-induced damage by activating SIRT3-PYCR1 to regulate proline metabolism to inhibit mitochondrial oxidative stress and improve cardiac function by inhibiting the p38MAPK-Ca^2+^ pathway and activating the AMPK-Parkin axis to inhibit necrosis (Fig. 6s).

## Discussion

As a member of the SIRT protein family, the structure of SIRT3 is similar to those of SIRT1, SIRT2 and SIRT5. Therefore, it is a challenge to design a targeted and specific small-molecule activator of SIRT3.^55–57^ In our previous studies, we demonstrated that SIRT3 is a potential therapeutic target and discovered a novel SIRT3 activator, 1-methylbenzylamino amiodarone (1-MA). 1-MA can increase SIRT3 deacetylation activity without changing the expression of SIRT3, but the specific binding of 1-MA to SIRT3 has not been verified.^58^ Therefore, in the next study, we identified the **L** pocket and the **U** pocket of SIRT3, which are different from those of SIRT1, SIRT2, SIRT3 and SIRT5, and demonstrated that these two pockets display better selectivity for SIRT3 than other SIRT family members. Accordingly, we successfully screened and discovered a targeted and specific small-molecule activator, ADTL-SA1215, by targeting the **U** pocket, which has a suitable ability to selectively activate SIRT3 and inhibit tumor cell proliferation and migration in triple-negative breast cancer models.^59^ However, high-dose ADTL-SA1215 showed considerable pulmonary toxicity in nontumorigenic mammary epithelial cell lines and triple-negative breast cancer mouse xenograft models.^60^ Additionally, High dose ADTL-SA1215 also showed cytotoxicity in rat cardiomyocytes H9c2, and could not exert myocardial protective effects in ISO induced cardiomyocyte injury (Supplementary Fig. 1). Thus, in this study, based on the **L** pocket, we screened and discovered a new targeted small-molecule activator of SIRT3, 2-APQC, which highly overlaps with the **L** pocket of SIRT3. Interestingly, unlike other SIRT3 positive modulators, such as resveratrol and melatonin, which activate SIRT3 by increasing its expression, 2-APQC was found to enhance SIRT3 deacetylase activity in both a time-dependent and dose-dependent manner without altering SIRT3 expression levels significantly. In addition, the binding stability of 2-APQC directly to SIRT1, SIRT2, SIRT4, SIRT5, SIRT6 and SIRT7 was poor, indicating that 2-APQC selectively activated SIRT3.

Moreover, we demonstrated that 2-APQC exhibited the ability to protect cardiomyocytes from ISO-induced hypertrophy and fibrosis by activating SIRT3. The mTOR/p70S6K pathway plays an important role in the development of pathological cardiac hypertrophy. The sustained activation of protein synthesis can lead to insufficient protein degradation and endoplasmic reticulum stress-induced cardiomyocyte apoptosis. Our results show that 2-APQC can reduce the protein expression level of the mTOR/p70S6K pathway. Typical TGF-β signal transduction mobilizes Smad2 and Smad3 transcription factors to promote gene expression, which induces fibroblasts to activate and differentiate into myofibroblasts that secrete extracellular matrix proteins and thus controlling fibrosis.^61^ SIRT3 reduces cardiac hypertrophy mediated by the activation of MnSOD2 and improves myocardial fibrosis by blocking the TGF-β/Smad3 pathway.^62,63^ In our study, we found that 2-APQC reduced the phosphorylation expression levels of TGF-β, JNK, Smad3 and Lox. The addition of mTOR/p70S6K pathway inhibitor PI3K/Akt/mTOR IN-2 and TGF-β/Smad3 pathway inhibitor BT173 further proves that 2-APQC improves fibrosis induced HF by blocking the mTOR/p70S6K pathway and TGF-β/Smad3 pathway to inhibit the development of cardiac fibrosis. In addition, we demonstrated that 2-APQC can ameliorate HF by relieving myocardial hypertrophy and inhibiting myocardial fibrosis in an ISO-induced HF model in vivo. To better evaluate the therapeutic effect of 2-APQC in vivo, we used two known small-molecule drugs, metoprolol and honokiol, as positive controls. Compared with the two positive controls, 2-APQC exhibited a comparable ability to protect the heart from myocardial hypertrophy and fibrosis.

*SIRT3* depletion promotes endothelial dysfunction, vascular hypertrophy, vascular inflammation, and terminal organ damage.^18^ In fact, several small-molecule compounds have been reported to improve heart failure by regulating SIRT3.^31–33,64–68^ LCZ696 induces upregulation of MnSOD2 through a SIRT3-dependent pathway to reduce myocardial oxidative stress and apoptosis in pressure-loaded HF.^31^ In addition, by activating SIRT3, Oroxylin A can promote the SIRT3-mediated expression of the *SOD2* gene by regulating the DNA binding activity of *FoxO3A* and increase the activity of *SOD2* by deacetylation.^32^ However, the effect of these aforementioned compounds on SIRT3 activation is not selective and as a result these compounds have poor therapeutic potential.^33^ Honokiol reduces cardiac hypertrophy by activating SIRT3.^69^ However, the therapeutic effects of magnolol on various diseases may be attributed to its antioxidant capacity of polyphenolic structures, rather than its positive regulatory ability on SIRT3. Additionally, it can reduce oxidative stress and apoptosis by activating the SIRT1-nuclear factor erythroid 2-related factor 2 signaling pathway. Moreover, the lipid kinase PIKfyve inhibitor STA-5326 prevents cardiomyocyte apoptosis and hypertrophy in obese mice by activating SIRT3. However, STA-5326 does not directly stimulate SIRT3 to treat myocardial hypertrophy.^70^ Resveratrol can activate SIRT3 and reduce cardiac hypertrophy and fibrosis in mice through the TGF-β/Smad3 pathway.^62^ However, these compounds are not satisfactory in terms of therapeutic effects or stimulation of SIRT3. Therefore, its anti-HF effect may not be produced by stimulating SIRT3. In our study, *SIRT3* knockout mouse model further decreased the EF% and FS% induced by ISO and eliminated the increase in EF% and FS% induced by 2-APQC. These results indicate that 2-APQC may improve ISO-induced cardiac dysfunction and exert cardioprotective effects by activating SIRT3 in heart failure model mice. The results of serum biochemical experiments also showed that knocking out *SIRT3* further increased AST levels and eliminated the decrease in AST levels induced by 2-APQC. These results indicate that 2-APQC may have a suitable protective effect on ISO-induced myocardial injury in mice by activating SIRT3. We used immunohistochemical testing on mouse heart tissue and found that after *SIRT3* knockout, myocardial hypertrophy and fibrosis were aggravated, and treatment with 2-APQC alleviated myocardial hypertrophy and myocardial cell fibrosis. The above experimental results indicate that 2-APQC can improve systolic function, inhibit myocardial injury, and alleviate hypertrophy symptoms and fibrosis in an ISO-induced heart failure rat model by activating SIRT3.

Based upon RNA-Seq analyses, we found that the SIRT3 activator 2-APQC could regulate the oxidative stress response and necrosis. We found that 2-APQC may activate SIRT3 to upregulate the expression of PYCR1, which can play a key role in apoptosis and oxidative stress.^71^ Indeed, 2-APQC can reduce mitochondrial and total ROS in H9c2 cells, which was increased by ISO. These findings indicate that 2-APQC may reduce ISO-induced oxidative damage by activating SIRT3 to upregulate PYCR1. As a key rate-limiting enzyme in proline metabolism, PYCR1 regulates the progression of disease mainly by regulating proline balance. In cancer, PYCR1 acts more like an oncogene, regulating proline metabolism in response to survival stress. In our study, we show for the first time that PYCR1 is significantly inhibited in ISO-induced HF rat models, accompanied by a significant reduction in proline. When SIRT3 was activated by 2-APQC, the expression of PYCR1 increased significantly, and the content of proline increased significantly, ROS decreased remarkably, indicating that SIRT3 can activate PYCR1 to resist ISO-induced mitochondria damage. However, after *SIRT3* knockout, 2-APQC could not activate PYCR1, which further proved that the activation of PYCR1 by 2-APQC was depended on SIRT3. After the deletion of *SIRT3*, the damage caused by ISO can also be significantly reduced by supplementing with proline, further confirming that PYCR1 is located downstream of SIRT3 and can be activated by SIRT3. Necrosis biomarkers are also closely related to inflammation and oxidative stress biomarkers, and myocardial cell necrosis may also stimulate the formation of the oxidative stress marker C-reactive protein.^72–76^ RIPK1, caspase 8 and FADD interact to form the RIPK1-FADD-caspase 8 complex, which leads to necrotic cell death, cardiac pathological remodeling and HF.^77,78^ In this study, we demonstrated that 2-APQC can regulate the AMPK-Parkin axis to inhibit the formation of the RIPK-FADD-caspase 8 complex and reduce the necrosis of cardiomyocytes. Moreover, we demonstrated that 2-APQC could inhibit necrosis by decreasing Ca^2+^ overload. When *SIRT3* was knockout, 2-APQC could not improve ISO-induced necrosis.

In summary, we discovered a new targeted SIRT3 activator, 2-APQC, using structure-based screening and verified that 2-APQC had remarkable efficacy in improving HF in vitro and in vivo. In the heart failure mouse model and *SIRT3* knockout mouse model, 2-APQC inhibited myocardial hypertrophy and relieved myocardial fibrosis by regulating the AKT-mTOR and TGF-β-Smad3 signaling pathways through SIRT3 activation. Moreover, 2-APQC protected cardiac cells from ISO-induced mitochondria damage by activating SIRT3 and then up-regulating PYCR1 to increase proline concent to inhibit mitochondrial oxidative stress, thereby improving cardiac function by inhibiting the p38MAPK-Ca^2+^ pathway and activating the AMPK-Parkin axis to inhibit necrosis. As mentioned above, these findings demonstrate that 2-APQC is a targeted SIRT3 activator as a a promising potential drug candidate that alleviates myocardial hypertrophy and fibrosis by regulating mitochondrial homeostasis for the future HF therapeutics.

## Materials and methods

### Cell culture

H9c2 rat cardiomyoblasts were purchased from ATCC (Virginia, USA). H9c2 cells were cultured in DMEM containing 10% fetal bovine serum (FBS, Biological Industries, Israel), 100 units/mL penicillin (HyClone), and streptomycin (HyClone) and grown in 37 °C incubators with a humidified 5% CO_2_ atmosphere.

### Plasmid transfection

The *SIRT3* and *PYCR1* plasmids were synthesized by Genechem(Shanghai, China). Mix an appropriate amount of plasmid DNA with Lipofectamine 3000 (ThermoFisher, L3000015) reagent to form a transfection complex. Add this complex onto pre-washed cells, dropwise, ensuring uniform distribution. Subsequently, incubate the cells in a cell incubator at 37 °C with 5% CO_2_ for 48 h.

### In vitro SIRT3 deacetylase activity assay

The SIRT3 activity test was conducted in accordance with the manufacturer’s instructions using a SIRT3 activity assay kit (Enzo Life Sciences, BML-AK557-0001). In short, recombinant human SIRT3 (BML-SE270) was incubated for 30 min at 37 °C with 10 µM fluorescent acetylated peptide substrate and 500 µM NAD^+^. Developer II/2 mM nicotinamide was added to stop the reaction, and the deacetyl-dependent fluorescent signal was developed at 37 °C. A CytoFluorII fluorescent plate reader (Perseptive Biosystems, Ex. 360 nm, Em. 460 nm) was then used to measure the fluorescence. Every experimental sample had the assay buffer’s fluorescence intensity deducted from it.

### SPR experiment

The instrument used for the interaction study of biomacromolecules is the Biacore X100 from Cytiva. The experiment involves the use of Biacore Control software 2.0.2 for experimental protocol setup and Biacore Evaluation software 2.0.2 for data analysis. Chip coupling begins with the use of EDC/NHS (Cytiva, BR-1006-33) in a 1:1 volume ratio. CM5 chips (Cytiva, BR-1005-30) are used to activate channels 1 and 2, with an activation time of 7 min. Switching to channel 2, ligand coupling is performed using acetic acid (Cytiva, BR100352) pH 5.0 to dilute the SIRT3 protein sample (Cloud Clone Corp, APE913Ra01), resulting in a coupling amount of 10 μg and a coupling signal of 5607.7 RU. Finally, switching back to channels 1 and 2, ethanolamine (Cytiva, BR-1006-33) is used for chip blocking, with a blocking time of 7 min. Preparation of running buffer and solvent calibration curve: The running buffer for small molecule samples is prepared using 1× PBS-P+ containing 5% DMSO. Take 105 mL of 10× PBS-P+ and dilute with deionized water to 1 L to obtain 1.05× PBS-P. Add DMSO according to the table to prepare 5% DMSO running buffer and 4.5%, 5.8% solvent calibration master solution. Replace the original running buffer in the left tray of the system with 1× PBS-P+ containing 5% DMSO and insert the corresponding inlet tube.2-APQC sample preparation: Dilute 20 mM 2-APQC stock solution 20 times with 1.05× PBS-P+ buffer without DMSO to obtain 1 mM small molecule in 1× PBS-P+ containing 5% DMSO (400 μL). Then, further dilute the analyte down threefold using the prepared running buffer to create 5 concentration gradients (each 200 μL), including 10 μM, 3.33 μM, 1.11 μM, 0.123 μM, 0.013 μM. Set an additional duplicate concentration with a zero concentration in between. Perform multiple-cycle kinetic measurements.

### CETSA assay

The CETSA assay is a method that can directly detect the target binding status in cells by detecting protein thermal stability changes.^79^ Here, after treatment with DMSO and 10 μM 2-APQC for 6 h, H9c2 cells were resuspended and centrifuged and then divided into seven parts. The cells were heated at 37, 41, 45, 49, 53, 57 and 61 °C for 3 min and then frozen and thawed for three cycles by using liquid nitrogen. The samples were centrifuged at 17,000×*g* for 20 min to collect soluble proteins, which were analyzed by Western blot analysis.

### Molecular docking and molecular dynamics simulations

Virtual screening and molecular docking: First, the Lipinski’s rule of five was used to filter 212 thousand compounds from the SPECS database, leaving 97 thousand tiny molecules. Second, using the Libdock module of Discovery Studio Software (version 3.5), the filtered SPECS library was quickly molecular docked in the pocket L(Thr150, Pro151, Phe157, and Glu323) of SIRT3 (PDB code:4FVT). The top 1000 hits were then screened. The top 5 compounds were chosen to undergo a 10-ns MD analysis after the CDOCKER module was used to accurately dock the 1000 hits.

### Cell viability assay

In 96-well plates, H9c2 cells were plated in triplicate and subjected to 24-h treatments with the specified doses of 2-APQC or other agents. After administering isoproterenol to the model for 48 h, 200 μL of complete media per well were mixed with 20 μL of 5 mg/mL MTT (3-(4,5-dimethylthiazol-2-yl)-2,5-diphenyltetrazolium bromide) (Sigma-Aldrich, St. Louis, MO, USA). The absorbance was measured at 490 nm using an ELISA reader (Bio-Rad, Hercules, CA, USA) 4 h later. To explore the impact of SIRT3 knockdown on the experiment, plasmid transfection was conducted 48 h prior to cell treatment.

### Immunofluorescence assay

The immunofluorescence test was carried out according to earlier instructions.^80^ To summarize, H9c2 cells were subjected to the following procedures: 4% paraformaldehyde fixation, 0.2% Triton X-100 permeabilization in PBS, 1% BSA blocking, and an overnight incubation at 4 °C with primary antibodies (1:500) diluted in PBS containing 1% BSA. The cells were sealed with glycerol and stained with Hoechst for 8 min in the dark after being incubated with the fluorescent secondary antibody for one hour at room temperature. The stained picture was then photographed using a positive fluorescence microscope (Zeiss, Germany). For the statistics of immunofluorescence images, each group had 3 independent copies, and each copy randomly selected 6 fields of view for statistics and then averaged them.

### Western blot analyses

Rat heart tissues or cells were lysed for protein lysate in RIPA buffer supplemented with a combination of phosphatase and protease inhibitors (CST, #5871). A BCA kit was used for quantitative analysis (Biosharp). On an SDS-polyacrylamide gel, the same quantity of total protein was loaded, separated, and subsequently transferred onto a PVDF membrane. Subsequently, the PVDF membrane was obstructed using TBS-T that included 5% dairy powder. The primary antibody was then left to incubate on the membrane for an additional night at 4 °C. Following three TBS-T washes, the blot was left to cure for 1 h at room temperature using the matching horseradish peroxidase-linked secondary antibody. The blot was seen using an improved luminol-based chemiluminescent substrate following three thorough washings with TBST. We performed western blot assays using specific antibodies, including SIRT3 (CST, 2627), JNK (CST, 9252), p-JNK^Thr183^/^Tyr185^ (CST, 4668), SMAD3 (CST, 9513), p-SMAD3^Ser423/425^ (CST, 9520), Akt (CST, 4691), p-Akt^Thr308^ (CST, 13038), mTOR (CST, 2972), p-mTOR^Ser2448^ (CST, 2971), PYCR1 (CST, 47935), p38MAPK (CST, 8690), p-p38MAPK^Thr180/Tyr182^ (CST, 4511), AMPK (CST, 5831), p-AMPK^Thr172^ (CST, 2535), acetylated lysine (Abcam, 9441), Ac-MnSOD (K68) (Abcam, ab137037), Ac-MnSOD (K122) (Abcam, ab214675), collagen I (Proteintech, 14695-1-AP), fibronectin (Abcam, ab2413), α-SMA (Proteintech, 55135-1-AP), TGF-β(Proteintech, 21898-1-AP), Lox (Proteintech, 17958-1-AP), Parkin (CST, 4211), RIPK3 (Proteintech, 17563-1-AP), Caspase-1 (Proteintech, 22915-1-AP), Caspase-8 (Proteintech, 13423-1-AP), and β-actin (CST, 5174).

### ROS, Ca^2+^ and PI measurements

ROS detection: Briefly, after drug treatment, H9c2 cells were collected and washed with PBS three times, and ROS staining solution was added at 1:1000. The cell suspension was treated with ROS staining solution for 30 min at 37 °C and shaken gently every three minutes. After removing the ROS staining solution by centrifugation, the cell suspension was PBS-washed three times. Ultimately, the cell suspension was assessed using flow cytometry at 488 nm for excitation and 525 nm for emission wavelengths.

Ca^2+^ detection: In short, following medication administration, H9c2 cells were collected and subjected to three PBS washes. After adding the lysis solution for three minutes, the mixture was centrifuged for three minutes at 14,000×*g*, and the supernatant was collected. After adding 150 μL of working solution and 50 μL of supernatant to 96-well plates, they were incubated in the dark for 10 min, and the absorbance at 575 nm was measured. Finally, using the standard curve, the Ca^2+^ concentration was calculated.

PI detection: Evaluate cell death in H9c2 cardiac cells using propidium iodide (Beyotime Biotechnology, ST511) flow cytometry. Wash cultured H9c2 cells with 1× PBS, collect and suspend them in PBS at an appropriate concentration. Add PI staining solution to the cell suspension and incubate for 20 min under light-protected conditions. Subsequently, employ a flow cytometer with excitation wavelength 535 nm and detection wavelength 617 nm to perform fluorescence detection.

### Analysis of serum biochemical indices

The LDH, AST, CK-MB and α-HBDH in plasma were measured by an automatic chemistry analyzer (Mindary BS-120; Mindray Bio-Medical Electronics Co., Ltd., Shenzhen, China). BNP levels were measured by an ELISA kit (Elabscience, E-EL-R0126c) according to the experimental instructions.

### Echocardiography/hemodynamic

For these measurements, experimental animals were anesthetized with 1.5% isoflurane (Aladdin) at four weeks, and cardiac function was assessed via echocardiography using an Acuson Sequoia C256 (Siemens) ultrasound machine. FS%, EF%, LVEDd and LVESd were measured as previously reported. After dissection, the weight of the rat heart and left ventricle were determined.

### Animals and experimental protocols

Male SD rats (male, 220–250 g) were purchased from Beijing Huafukang Biotechnology Co., Ltd. *SIRT3* knockout model mice and wild type mice were purchased from Yangzhou Youdu Biotechnology Co., Ltd. The study’s animal experiments were all carried out in accordance with institutional policies. The rats were divided into 7 groups as follows (*n* = 6–9): CON, ISO, 2-APQC (L) + ISO, 2-APQC (M) + ISO, 2-APQC H + ISO, MET and HON. All of the groups except the CON group were injected subcutaneously with 5 mg/kg/d ISO for 2 weeks. Then, the rats were examined by echocardiography to evaluate HF modelling. After the model was successfully established, 3 mL/kg/d normal saline was fed to the CON and ISO groups. In the 2-APQC (L) + ISO, 2-APQC (M) + ISO and 2-APQC H + ISO groups, 10 mg/kg/d, 20 mg/kg/d and 30 mg/kg/d of 2-APQC was intragastrically administered. The MET and HON groups were given 10 mg/kg/d honokiol and metoprolol, respectively. The markers discussed in this article, such as heart mass index, histology, and echocardiography, were assessed 4 weeks later.

*SIRT3* knockout model mice and wild type mice were purchased from Yangzhou Youdu Biotechnology Co., Ltd. All animal experiments performed in the study were conducted in compliance with institutional guidelines. The mice were divided into 6 groups as follows (*n* = 6–9): WT-CON, WT-ISO, WT-ISO + 2-APQC, SIRT3-KO-CON, SIRT3-KO-ISO, SIRT3-KO-ISO + 2-APQC. All of the groups except the CON group were intraperitoneal injected subcutaneously with 30 mg/kg ISO for 2 weeks. Then, the mice were examined by echocardiography to evaluate HF modelling. After the model was successfully established, 3 mL/kg/d normal saline was fed to the CON and ISO groups. In the ISO + 2-APQC group, 42 mg/kg of 2-APQC was intraperitoneal injection administered. The markers discussed in this article, such as heart mass index, histology, and echocardiography, were assessed 4 weeks later.

### Immunohistochemistry assay

Briefly, the organs were heated with antigen retrieval buffer (pH 8.0) or citrate buffer (pH 6.0). After that, the tissues were divided into sections, and those sections were incubated at 37 °C for 30 to 40 min with the matching antibody. After 30 min of incubation, the sections were colored with diaminobenzidine solution and secondary antibodies coupled with HRP polymer. Image-Pro Plus 6.0 software (Media Cybernetics, Rockville, MD, USA) was used to analyze the stained images.

### Hematoxylin and Eosin staining

The tissue was embedded into a wax block and sectioned, the sections were stained for 5 min with hematoxylin, and tap water was used to wash for 1 min. Next, hydrochloric acid and ethanol were used to differentiate for 30 s. The piece was cleaned for 5 min with warm water, then immersed in eosin solution for 2 min. Regular dehydration was then performed, and resin was sealed.

### Sirius red staining

The sections were soaked in xylene, in two changes, for 10–20 min each. Gradient elution with anhydrous ethanol and rinsing with distilled water was performed. The sections were rinsed twice with PBS. Excess water was removed from the tissue sections, Sirius red staining solution was added dropwise, and staining was performed at room temperature for 1 h. The sections were rinsed twice with PBS and flowing water to remove any remaining staining solution on the surface of the sections. The sections were soaked in a gradient of 70% alcohol to anhydrous ethanol. After soaking in xylene for 10–20 min to dehydrate and turn transparent, the pieces were taken out, given a brief period of drying time, and sealed with neutral gum. Images were captured under a microscope and analyzed.

### WGA staining

After paraffin sections were dewaxed, they were placed in EDTA solution for antigen repair. After adding the WGA staining solution, the mixture was incubated for 30 min at 37 °C. After washing with PBS solution three times, the sections were dried. After adding the DAPI staining solution, the mixture was allowed to sit at room temperature for ten minutes. The sections were dried and sealed with anti-quenching agent. Ultimately, pictures were examined and gathered using fluorescence microscopy, and ImageJ was used to calculate the cell area.

### Masson staining

The masson staining procedure involves dewaxing and rehydrating heart sections through a series of xylene and ethanol steps. Subsequently, the sections are fixed in Bouin solution, followed by rinsing until the disappearance of yellow color. Staining is then performed using Weigert’s iron hematoxylin and Mayer’s hematoxylin, with differentiation in acidified ethanol and subsequent rinsing. Additional staining steps include Ponceau S fuchsin, phosphomolybdic acid treatment, and aniline blue application. After treating with weak acid, rapid dehydration, and xylene clearing, the sections are sealed with neutral gum on glass slides. Observation under a microscope focuses on collagen fibers (blue), muscle fibers (red), and cell nuclei. The mounted sections are affixed to glass slides for detailed examination.

### Proline (PRO) detection

Utilizing the PRO Detection Kit (Beijing Leagene Biotechnology, TC2161), appropriate H9c2 cell lysate is prepared using PRO Lysis buffer (1×) for homogenization. Following the kit instructions, specific solutions (distilled water, proline standard, proline extraction solution, PRO Assay buffer, and ninhydrin color developer) are added. After thorough mixing, the solution is subjected to a 30-min boiling water bath, resulting in a red-colored solution. Rapid cooling is performed, followed by the addition of toluene or xylene, shaking for 30 s, and allowing for a brief settling period. After centrifugation, the supernatant is transferred to a 96-well plate, and absorbance is measured at 520 nm using a spectrophotometer.

### RNA-seq and bioinformatic analysis

Trizol was used for the total RNA extracted, and the pair-ended sequencing of the Illumina Hiseq platform was used for all samples. Several R packages were used to analyze data and visualize the results. The DESeq2 package was performed to calculate the differentially expressed genes between the groups, and the genes with Log2(FC) > 1 and *P* value < 0.05 were regarded as upregulated genes, while the genes with Log2(FC) < −1 and *P* value < 0.05 were regarded as downregulated genes. Volcano plots of differentially expressed genes were generated via the ggplot2 package. The candidate genes were analyzed using the heatmap package to reflect the degree of differential expression. The clusterProfiler and org.Hs.eg.db packages were used to analyze the biological function of the differentially expressed genes, and the enrichment results were completed via the ggplot2 package. The SIRT3 protein‒protein interaction network was constructed using PrePPI (http://bhapp.c2b2.columbia.edu/PrePPI). Gene Ontology (http://geneontology.org) and KEGG (http://www.kegg.jp/kegg/) enrichment analyses were combined to find the key pathways enriched in the differentially expressed genes in the protein‒protein interaction (PPI) network. PPI network visualization was completed using Cytoscape software (version 3.7.2).

### Statistical analyses

All experiments were conducted independently at least 3 times, and the obtained data was represented as mean ± SEM. Data analysis was conducted using GraphPad Prism 8.0, and univariate analysis of variance and *t*-test were used for statistical comparison. ns, no significance; *p < 0.05, **p < 0.01, ***p < 0.001, ****p < 0.0001; ^#^*p* < 0.05, ^##^*p* < 0.01, ^###^*p* < 0.001, ^####^*p* < 0.0001.

### Supplementary information

SUPPLEMENTAL MATERIA

## Data Availability

The authors confirm that the data supporting the findings of this study are available within the article. The raw data of RNA sequencing have been deposited, please find it in the GEO database (No. GSE260489, https://www.ncbi.nlm.nih.gov/geo/). All experimental materials (cells, compounds, plasmids, etc.) will be available from the corresponding author upon reasonable request.
