# Supplementary material for: 2-APQC, a small-molecule activator of Sirtuin-3 (SIRT3), alleviates myocardial hypertrophy and fibrosis by regulating mitochondrial homeostasis
Source: Signal Transduct Target Ther. 2024 May 15;9:133. doi: 10.1038/s41392-024-01816-1 (PMC11094072; doi:10.1038/s41392-024-01816-1)
Supplement: Supplementary file 1 — SUPPLEMENTAL MATERIA [file 41392_2024_1816_MOESM1_ESM.docx]

Supplementary Materials for

**2-APQC，a small-molecule activator of Sirtuin-3 (SIRT3), alleviates myocardial hypertrophy and fibrosis by regulating mitochondrial homeostasis**

Fu Peng^1#^, Minru Liao^1#^, Wenke Jin^2#^, Wei Liu^1#^, Zixiang Li^2#^, Zhichao Fan^1,3^, Ling Zou^1,4,^, Siwei Chen^2,4^, Lingjuan Zhu^5^, Qian Zhao^3^, Gu Zhan^3^, Liang Ouyang^1^, Cheng Peng^3^, Bo Han^3^*, Jin Zhang^4^*, Leilei Fu^2^*

Corresponding to: [hanbo@cdutcm.edu.cn](mailto:hanbo@cdutcm.edu.cn);

zhangjin1989@szu.edu.cn;

leilei_fu@163.com

**This PDF file includes:**

Supplementary Fig. 1 to 4

Supplementary Table 1, 2

Original and uncropped films of Western blots


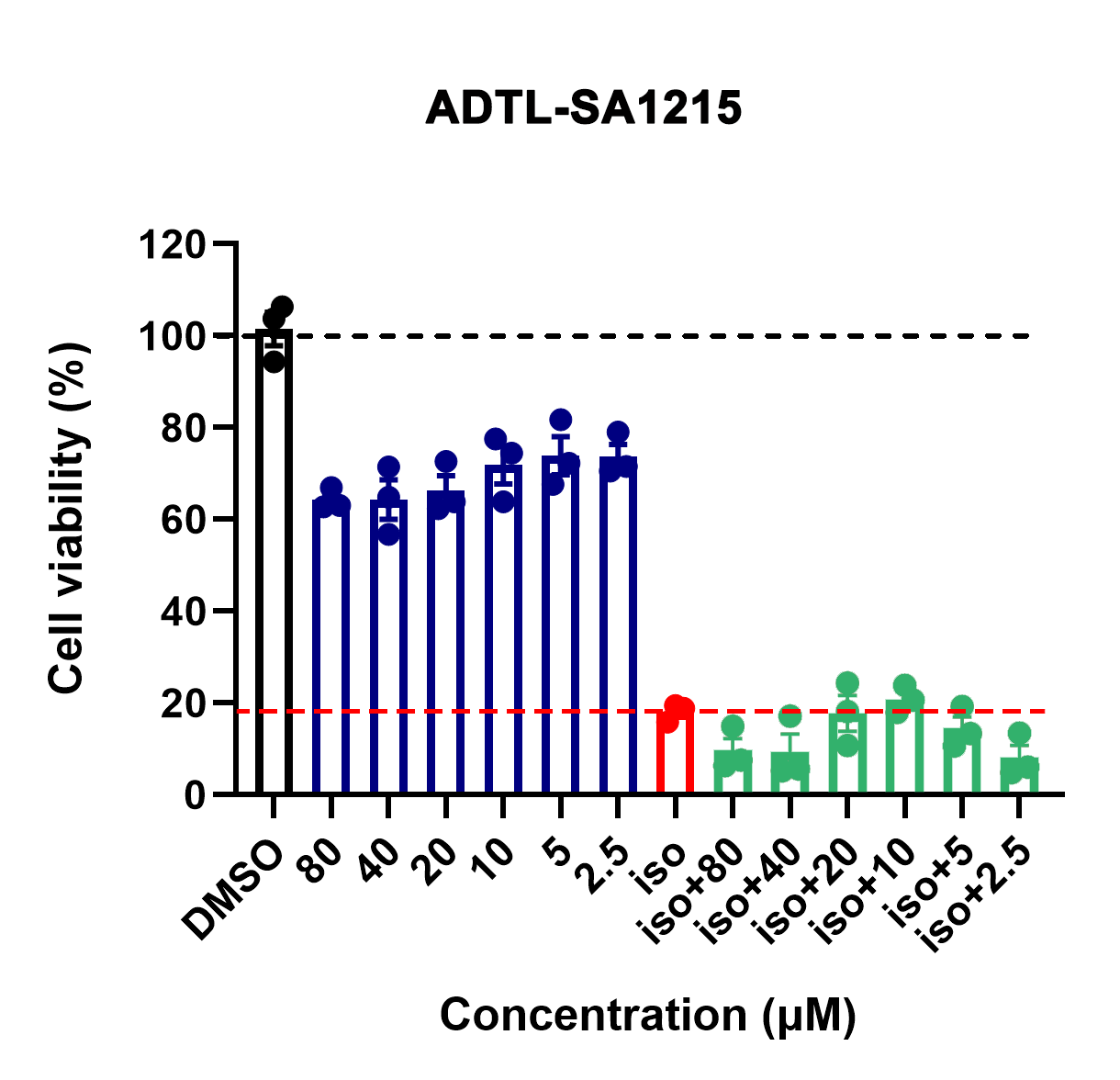


**Supplementary Fig. 1** Cytotoxicity of ADTL-SA1215 on H9c2 cells and protective effect against isoproterenol (ISO)-induced injury.


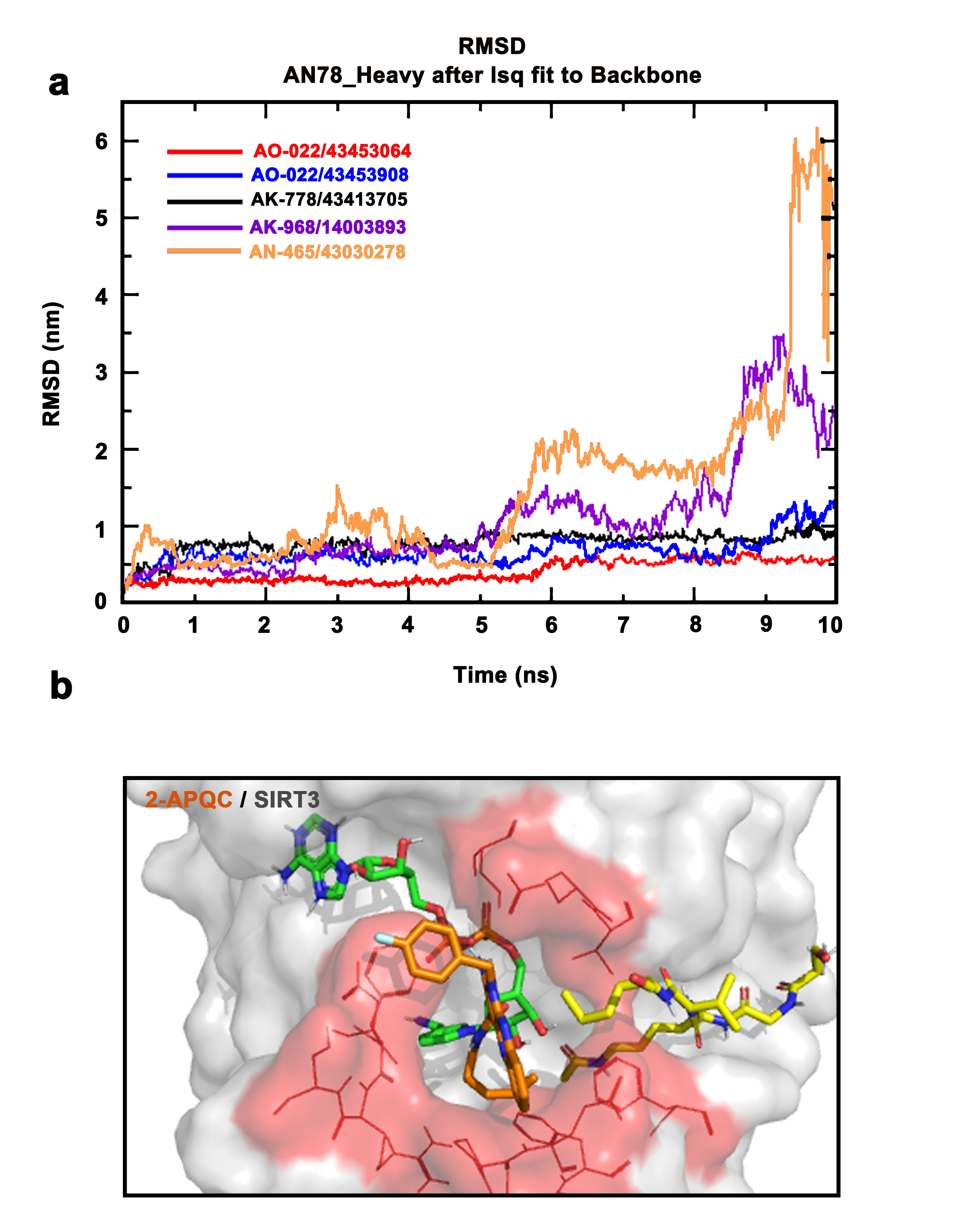


**Supplementary Fig. 2** a, Dynamic simulation of 5 candidates with SIRT3. b, Molecular docking of 2-APQC and Pocket **L** of SIRT3.





**Supplementary Fig. 3 a** Western blot analysis of SIRT1, SIRT2, SIRT3 and SIRT5 protein expression in H9c2 cells treated with 2-APQC for 24 h. **b** CETSA assay detected the thermal stability of SIRT1, SIRT2 and SIRT5 in H9c2 cells treated with 2-APQC. **c** Cellular thermal shift assay detected the thermal stability of SIRT4, SIRT6 and SIRT7 in H9c2 cells treated with 2-APQC. **d** Western blot analysis of acetylated-lysine, acetylated MnSOD2 at K68 and K122 protein expression in H9c2 cells treated with 2-APQC for 6 h, 12 h, 24 h and 48 h. β-actin was measured as a load control.


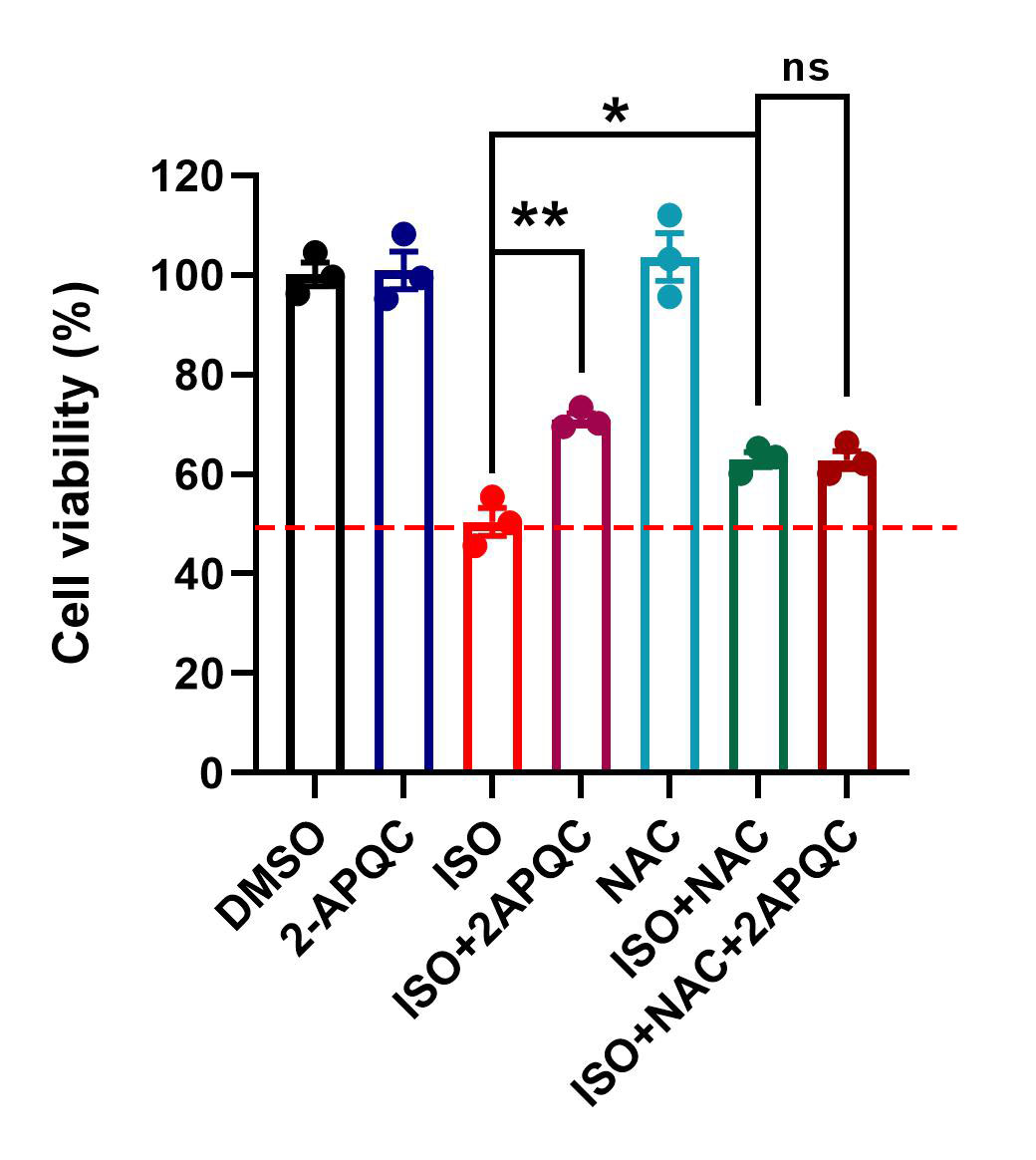


**Supplementary Fig. 4** Detection of the effect of NAC inhibition on ROS production on the cell viability of H9c2 cells by ISO and 2-APQC.

**Supplementary Table 1** The top 20 compounds based upon SIRT3 **Pocket L** screening

| **No.** | **Compound ID** | **SMILES** | **CDOCKER mode** | **CDOCKER ENERGY （Kcal/mol）** | **CDOCKER INTERACTION ENERGY （Kcal/mol）** |
| --- | --- | --- | --- | --- | --- |
| **1** | AN-979/41713880 | COc4ccccc4(OCC(=O)​Nc3ccc(Cc2ccc(NC(=O) COc1ccccc1(OC))c(O)c2)cc3(O)) | 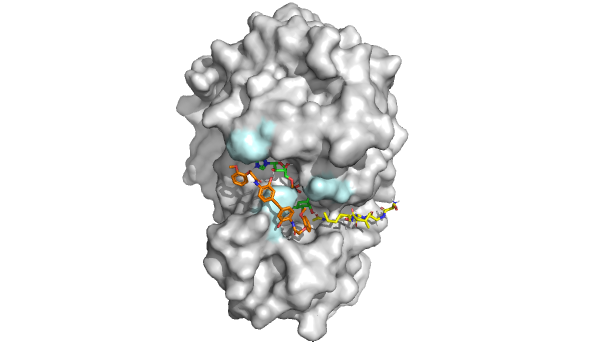 | -28.5441 | -48.2522 |
| **2** | AN-465/43030278 | [H] N(CCc1ccc(cc1) S(N) (=O)=O) Cc2ccc4c(c2) c3ccccc3n4(CC) | 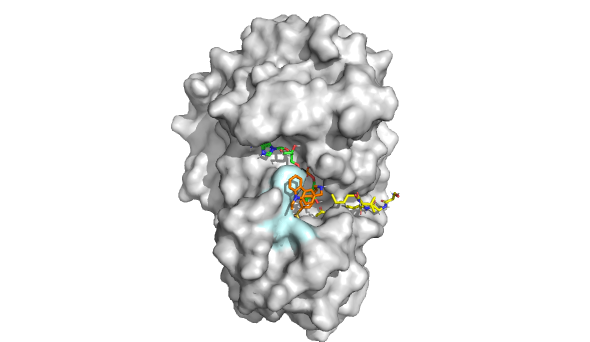 | -25.8471 | -35.3061 |
| **3** | AP-263/41946485 | NC(=O)C1CCN(CC1)CC(O) COc2ccc(cc2)OCC(O) CN3CCC(CC3)C(N)=O | 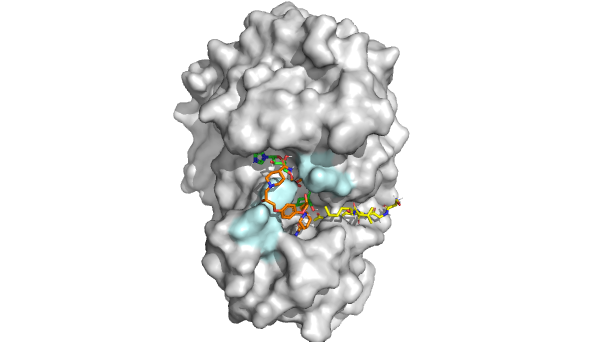 | -21.9744 | -51.5037 |
| **4** | AG-690/11203395 | CCOC(=O)CCSc3nc1c(C(=O)NC(=O) N1C)n3(CC(O)COc2ccc(cc2)Br) | 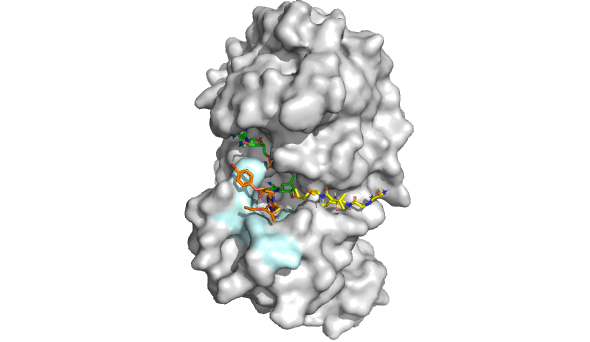 | -27.1837 | -38.4481 |
| **5** | AP-263/43417788 | CC(O)CNS(=O)(=O)c1ccCc(cc1Cl)Cl | 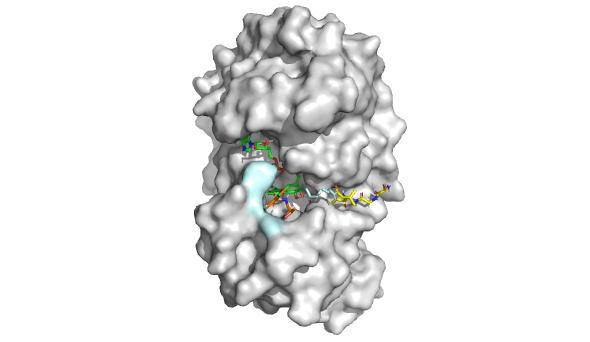 | -24.1083 | -28.3599 |
| **6** | AG-690/40112625 | CCOc4cccc(C=Nc1ccc(c(O)c1) c2nc3ccCccc3(o2))c4(O) | 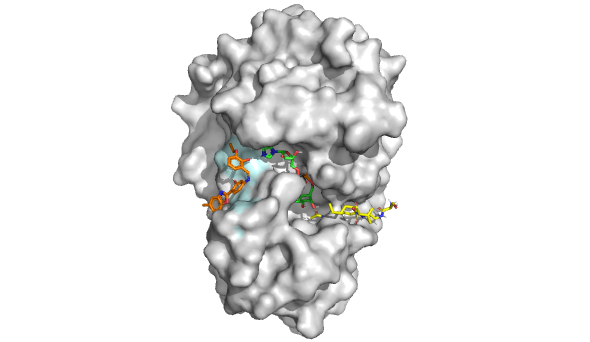 | -22.0239 | -35.4743 |
| **7** | AK-968/14003893 | CCOc1ccc(cc1) OCc2cccc(c2) C(=O) NCc3cccnc3 | 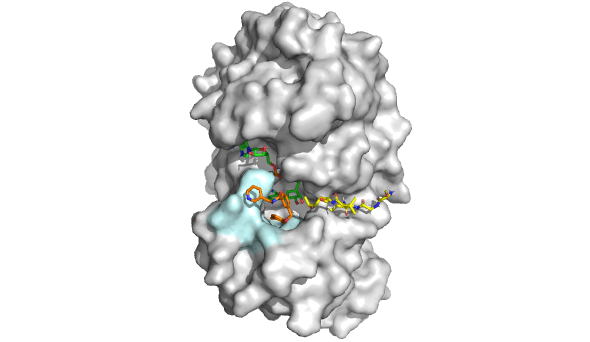 | -25.0156 | -37.0482 |
| **8** | AP-263/42850284 | COc1cc(OC)c(cc1S(N)(=O)=O) S(N)(=O)=O | 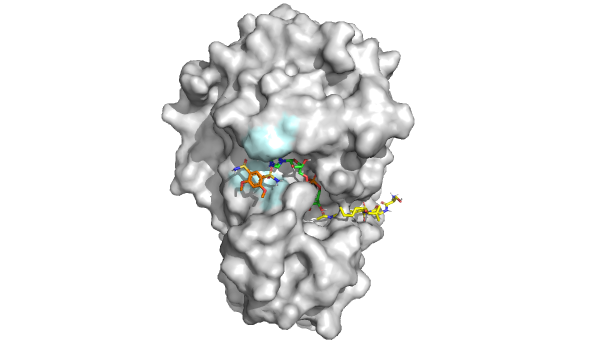 | -23.5351 | -27.9946 |
| **9** | AT-057/43485503 | Cc4ccccc4(Nc2c(nc1ncccn12)c3ccc(O) c(O)c3) | 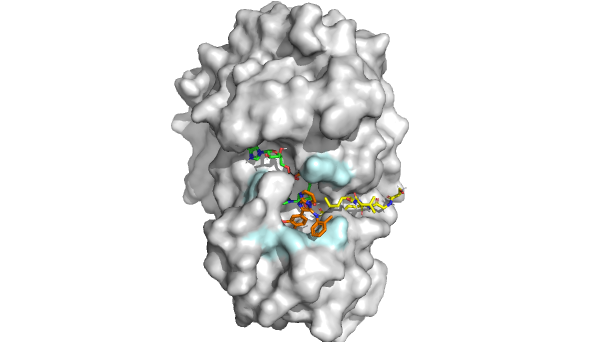 | -19.3919 | -30.0356 |
| **10** | AE-562/43458218 | Nc1ccc2C(=O)c3ccc(O)c(O)c3(C(=O) c2(c1)) | 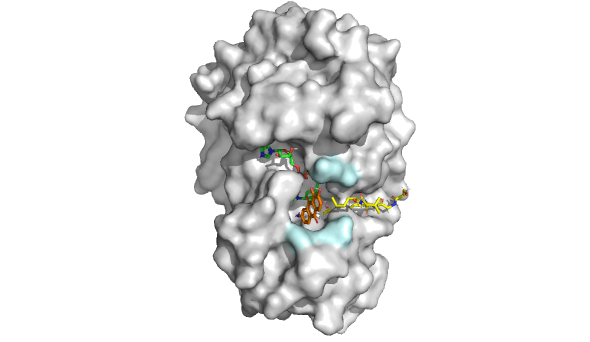 | -23.03 | -28.4951 |
| **11** | AP-970/43506970 | CC(=O)Nc2cc(N)ccC c2(OCc1ccc(cc1Cl)Cl) | 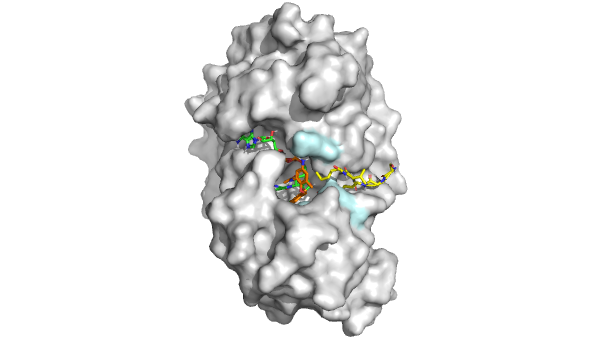 | -22.3721 | -31.5956 |
| **12** | AK-968/09126008 | OC(=O)c1ccc(cc1)NC(=O)C2CCCCC2(C(O)=O) | 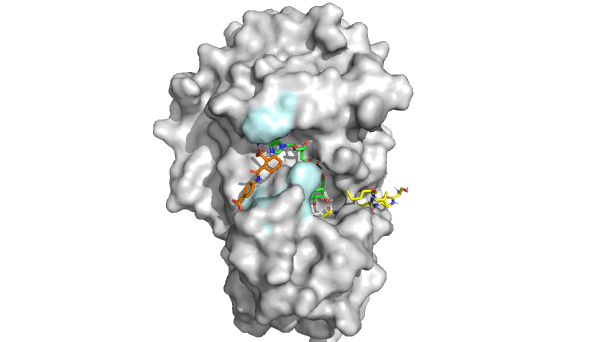 | -23.6429 | -31.8888 |
| **13** | AE-641/42119771 | OC(=O)c1cc(ccc1(O))S(=O)(=O) Nc2cccc(c2)Cl | 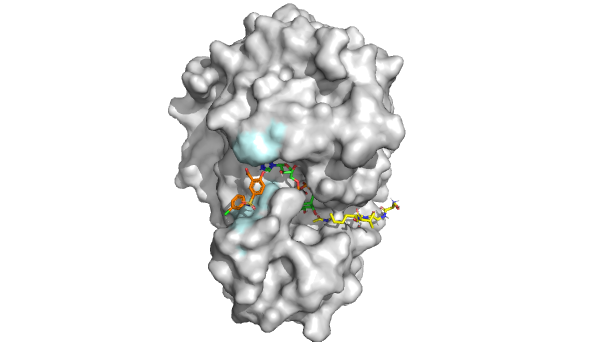 | -21.2804 | -32.4215 |
| **14** | AO-022/43453908 | COc3ccc4CC=C(CCC(=O) NCC Cc1c[nH] c2ccccc12) C(=O) Oc4(c3) | 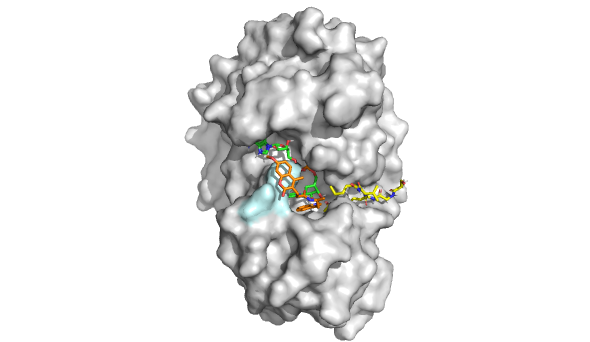 | -15.8137 | -37.9332 |
| **15** | AE-641/04635016 | NC(=O)c2cccnc2(Nc1cccc(c1)CF FF) | 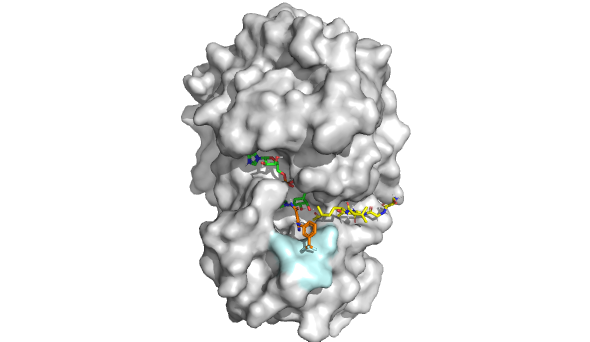 | -15.4392 | -26.5311 |
| **16** | AO-022/43453064 | CCCCCNC(=O) c3c(N) n(Cc1cccF cc1) c2nc4ccccc4(nc23) | 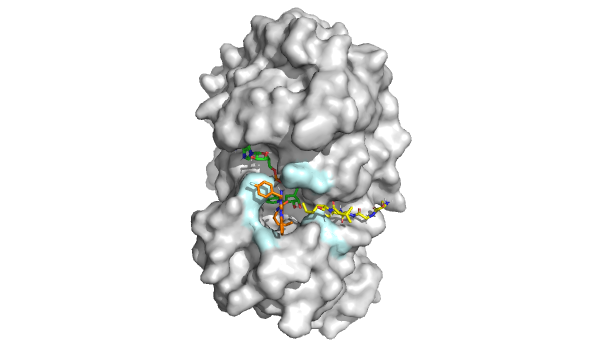 | -15.2084 | -37.9332 |
| **17** | AG-205/15155107 | COc1cc(cc(OC)c1(OC))C(=O) Nc2ccc(cc2)OCC(N)=O | 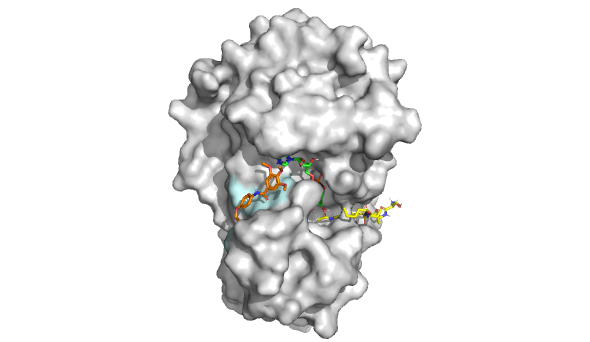 | -17.1871 | -41.8824 |
| **18** | AK-778/43413705 | CCOc1ccc(cc1) C(=O) CC4(O) (C(=O) N(Cc2cccC cc2) c3ccc(cc34) Cl) | 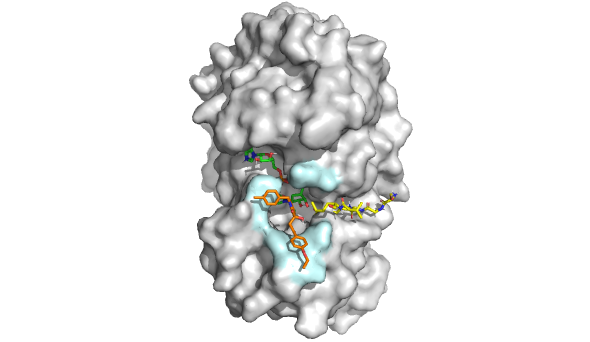 | -12.8412 | -35.9074 |
| **19** | AE-562/12222311 | CN(NOC1OC(CO)C(O)C(O)C1(O)) c2ccccc2 | 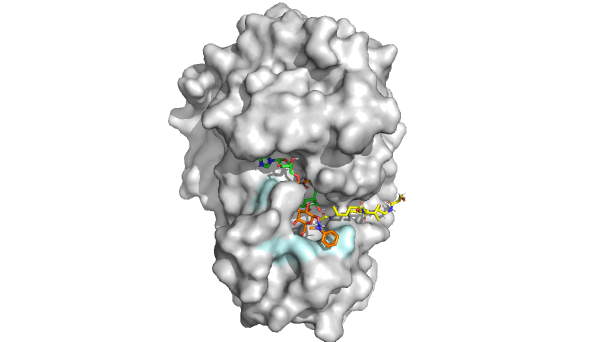 | -5.6413 | -31.16 |
| **20** | AK-968/12265001 | CCc3ccc4NC(=O)C(c1ccc(O)cCc1)(c2ccc(O)cCc2)c4(c3) | 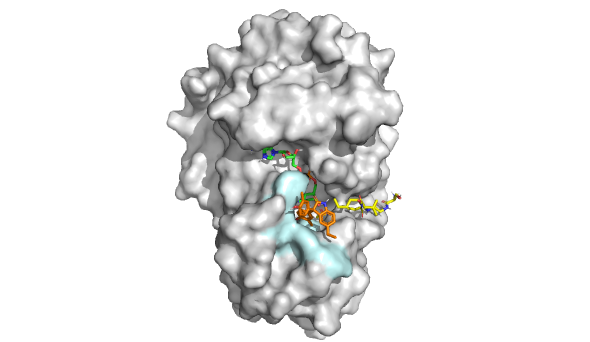 | -11.9243 | -36.4647 |

**Supplementary Table 2** The top 50 changed genes of RNA-seq.

| **Gene_ID** | **Symbol** | **description** | **Mean-control** | **Mean-iso** | **Mean-iso+2-APQC** | **control_vs_iso_FoldChange** | **iso+2-APQC_vs_iso_FoldChange** |
| --- | --- | --- | --- | --- | --- | --- | --- |
| ENSRNOG00000016243 | Casq2 | calsequestrin 2 | 14.65591877 | 15.31930588 | 14.7881411 | 1.583796646 | 0.691995814 |
| ENSRNOG00000012991 | Adgra2 | adhesion G protein-coupled receptor A2 | 12.87307222 | 12.29450294 | 12.73116825 | 0.669627519 | 1.35347225 |
| ENSRNOG00000012802 | Tenm3 | teneurin transmembrane protein 3 | 12.39380736 | 10.56197384 | 11.23492356 | 0.280907389 | 1.594329386 |
| ENSRNOG00000016552 | Hmgcs1 | 3-hydroxy-3-methylglutaryl-CoA synthase 1 | 12.21871414 | 13.0673579 | 12.31194777 | 1.800807242 | 0.592377959 |
| ENSRNOG00000046168 | Ppm1l | protein phosphatase, Mg2+/Mn2+ dependent, 1L | 11.27439468 | 9.407072853 | 10.0034315 | 0.274081751 | 1.511895734 |
| ENSRNOG00000029134 | Adgrl1 | adhesion G protein-coupled receptor L1 | 11.26426098 | 10.01630925 | 10.41017036 | 0.421045562 | 1.313905133 |
| ENSRNOG00000019476 | Agap1 | ArfGAP with GTPase domain, ankyrin repeat and PH domain 1 | 11.20950541 | 6.92133563 | 7.553204329 | 0.05118377 | 1.549570827 |
| ENSRNOG00000048922 | LOC100909664 | centrosomal protein of 170 kDa-like | 11.17139849 | 8.359026933 | 8.760374123 | 0.142361253 | 1.320740644 |
| ENSRNOG00000007315 | Thoc2 | THO complex 2 | 11.01540566 | 9.678389076 | 10.08384941 | 0.395838381 | 1.324511463 |
| ENSRNOG00000011700 | Ptprm | protein tyrosine phosphatase, receptor type, M | 10.9455291 | 7.810936297 | 8.432662916 | 0.113865863 | 1.538715616 |
| ENSRNOG00000004165 | Prkd1 | protein kinase D1 | 10.27463692 | 7.242398919 | 7.931115403 | 0.122237767 | 1.611848873 |
| ENSRNOG00000009329 | Nr1d1 | nuclear receptor subfamily 1, group D, member 1 | 10.14441644 | 11.7300401 | 11.20307469 | 3.00137516 | 0.694012997 |
| ENSRNOG00000020776 | Dhcr7 | 7-dehydrocholesterol reductase | 9.797798639 | 10.79255589 | 10.235593 | 1.992745193 | 0.679731607 |
| ENSRNOG00000022812 | Ercc5 | ERCC excision repair 5, endonuclease | 9.737243024 | 8.734412052 | 9.16785139 | 0.499019822 | 1.350449169 |
| ENSRNOG00000011739 | Dennd4a | DENN domain containing 4A | 9.612653042 | 7.86387268 | 8.279688339 | 0.297553221 | 1.334052699 |
| ENSRNOG00000033697 | Casp4 | caspase 4 | 9.593411677 | 9.066944043 | 9.566271963 | 0.694252497 | 1.413554904 |
| ENSRNOG00000031197 | F8 | coagulation factor VIII | 9.567473183 | 8.61889984 | 9.027420203 | 0.518144593 | 1.327323804 |
| ENSRNOG00000012184 | Urgcp | upregulator of cell proliferation | 9.523302315 | 8.489218492 | 8.89629383 | 0.488325891 | 1.325995003 |
| ENSRNOG00000018766 | Rrp8 | ribosomal RNA processing 8, methyltransferase, homolog (yeast) | 9.467039677 | 7.981024647 | 8.593322067 | 0.356997276 | 1.528691637 |
| ENSRNOG00000012349 | Mesdc1 | mesoderm development candidate 1 | 9.419954072 | 7.58098869 | 8.03099012 | 0.27952217 | 1.366041611 |
| ENSRNOG00000047741 | E2f2 | E2F transcription factor 2 | 9.27971672 | 7.079905142 | 7.744162913 | 0.217666067 | 1.58475275 |
| ENSRNOG00000008409 | Myo1f | myosin IF | 9.227035656 | 7.5310874 | 8.118174654 | 0.308651722 | 1.502210778 |
| ENSRNOG00000002053 | Fras1 | Fraser extracellular matrix complex subunit 1 | 9.18733496 | 4.770686668 | 6.080392979 | 0.046822692 | 2.478910717 |
| ENSRNOG00000036682 | Pycr1 | pyrroline-5-carboxylate reductase 1 | 9.17831061 | 8.583385082 | 9.036743448 | 0.662078631 | 1.369223887 |
| ENSRNOG00000008144 | Irf1 | interferon regulatory factor 1 | 9.17230495 | 10.28001703 | 9.761440064 | 2.155036167 | 0.69806004 |
| ENSRNOG00000007489 | Zfp41 | zinc finger protein 41 | 9.053914159 | 8.013343266 | 8.516853163 | 0.486135066 | 1.417658357 |
| ENSRNOG00000011704 | Fbxo34 | F-box protein 34 | 9.022655812 | 9.434047788 | 8.769063411 | 1.329968408 | 0.630695534 |
| ENSRNOG00000033433 | Csrnp1 | cysteine and serine rich nuclear protein 1 | 9.009213205 | 11.3452648 | 10.56478954 | 5.049188695 | 0.58217498 |
| ENSRNOG00000033809 | Mlh1 | mutL homolog 1 | 9.007455653 | 8.006927138 | 8.417671332 | 0.499816864 | 1.329371375 |
| ENSRNOG00000013844 | Fam172a | family with sequence similarity 172, member A | 8.974791608 | 7.124147855 | 7.706179484 | 0.277268619 | 1.496955805 |
| ENSRNOG00000001750 | Chrd | chordin | 8.928014201 | 7.793635598 | 8.308850252 | 0.455531081 | 1.429206772 |
| ENSRNOG00000017072 | Slc16a14 | solute carrier family 16, member 14 | 8.866433314 | 7.930498454 | 8.522455041 | 0.522703651 | 1.507289552 |
| ENSRNOG00000012775 | Ccdc127 | coiled-coil domain containing 127 | 8.837835743 | 7.623193054 | 8.216343577 | 0.430879782 | 1.508537461 |
| ENSRNOG00000010227 | Gpatch2l | G patch domain containing 2-like | 8.69361409 | 7.943920087 | 8.395366711 | 0.594729687 | 1.367410704 |
| ENSRNOG00000028390 | Hhipl1 | HHIP-like 1 | 8.459470841 | 7.51617974 | 8.349260171 | 0.52004519 | 1.781485117 |
| ENSRNOG00000056153 | Fam46b | family with sequence similarity 46, member B | 8.42374029 | 8.924317497 | 8.376237989 | 1.414779487 | 0.683929959 |
| ENSRNOG00000013641 | Myo7a | myosin VIIA | 8.416444748 | 7.368892198 | 8.144571704 | 0.483788188 | 1.711996209 |
| ENSRNOG00000016677 | Csnk1g3 | casein kinase 1, gamma 3 | 8.349645702 | 6.963237906 | 7.627286273 | 0.382516054 | 1.584522742 |
| ENSRNOG00000049714 | Asap3 | ArfGAP with SH3 domain, ankyrin repeat and PH domain 3 | 8.342011361 | 7.723405492 | 8.184242569 | 0.6513 | 1.376340163 |
| ENSRNOG00000014061 | Dusp5 | dual specificity phosphatase 5 | 8.23328435 | 10.60801517 | 9.840196181 | 5.186390458 | 0.587304667 |
| ENSRNOG00000000413 | Pln | phospholamban | 7.866576576 | 9.539492347 | 8.622725704 | 3.188583741 | 0.529694839 |
| ENSRNOG00000015669 | Kctd11 | potassium channel tetramerization domain containing 11 | 7.419069925 | 6.028608791 | 6.921671608 | 0.381442861 | 1.857114564 |
| ENSRNOG00000057945 | Fancg | Fanconi anemia, complementation group G | 7.342075128 | 5.231961246 | 6.175791019 | 0.231628731 | 1.923627923 |
| ENSRNOG00000011136 | Osr2 | odd-skipped related transciption factor 2 | 7.326968967 | 8.784015297 | 8.070753406 | 2.745457027 | 0.609939523 |
| ENSRNOG00000016612 | Trim36 | tripartite motif-containing 36 | 7.025050523 | 8.528214343 | 7.947868866 | 2.834636651 | 0.668803602 |
| ENSRNOG00000042826 | Zfp52 | zinc finger protein 52 | 6.973203095 | 5.865576991 | 6.629122753 | 0.46405699 | 1.697657895 |
| ENSRNOG00000046443 | Mthfsd | methenyltetrahydrofolate synthetase domain containing | 6.787793808 | 5.417215257 | 6.381553703 | 0.386736128 | 1.951168599 |
| ENSRNOG00000005450 | Lsm11 | LSM11, U7 small nuclear RNA associated | 6.759494148 | 5.955400941 | 6.710499788 | 0.572721946 | 1.687747225 |
| ENSRNOG00000023991 | Rab20 | RAB20, member RAS oncogene family | 6.737477954 | 8.938076325 | 8.284420382 | 4.596699549 | 0.635667422 |
| ENSRNOG00000061096 | Rn7sl1 | RNA, 7SL, cytoplasmic 1 | 6.625013798 | 10.35389061 | 7.67114545 | 13.25878631 | 0.155744685 |

**Original and uncropped films of Western blots**

**Figure 1**


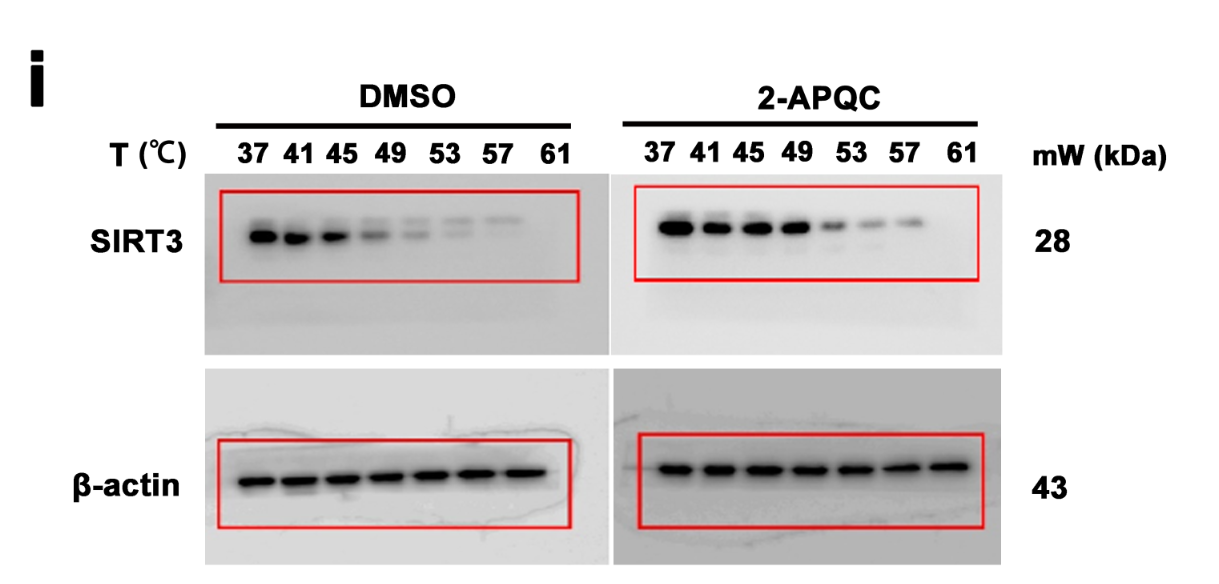


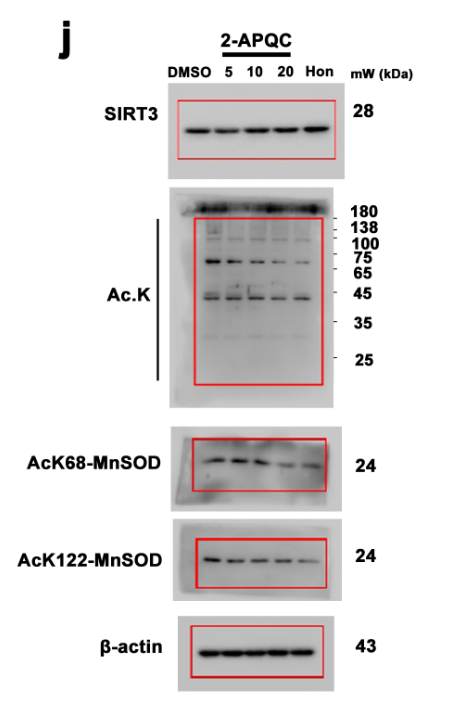


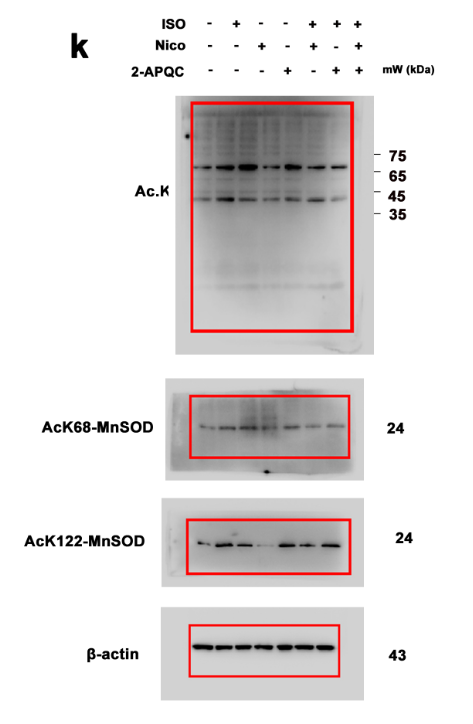


**Figure 2**


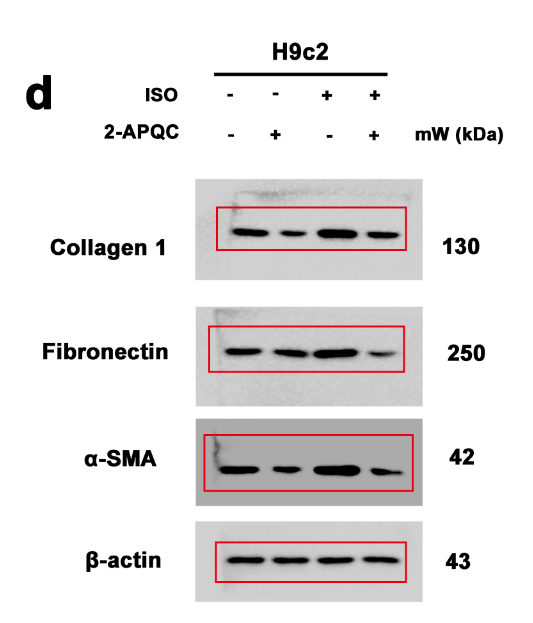


**Figure 3**

**
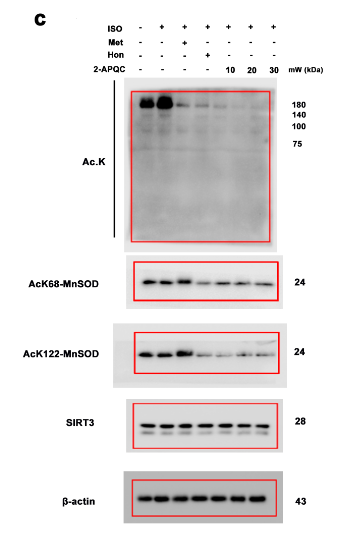
**

**
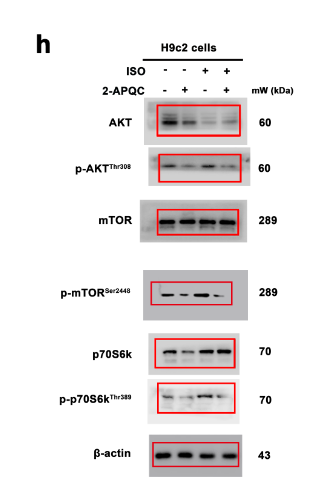
**

**
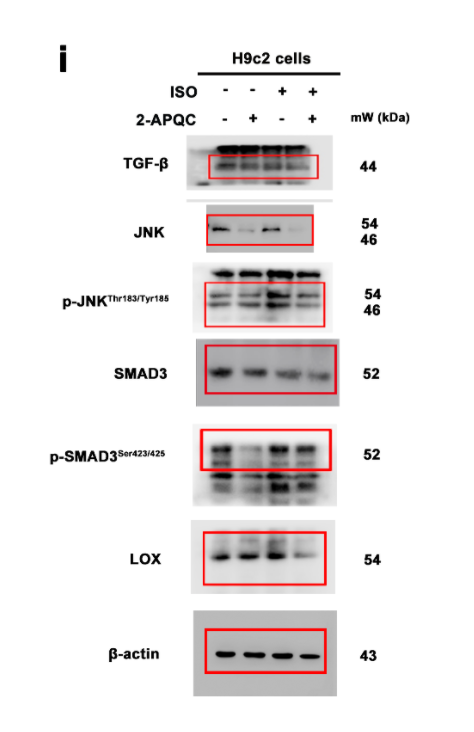
**

**
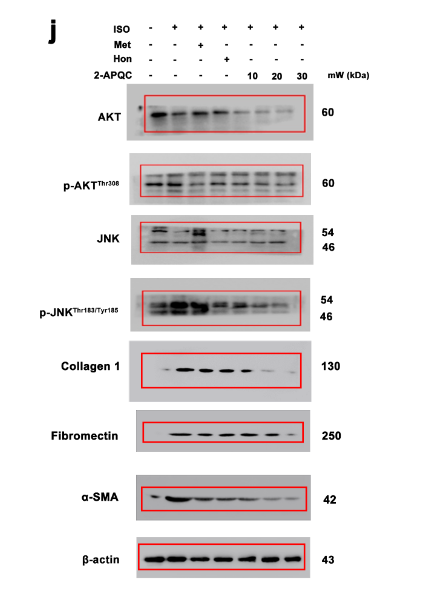
**

**
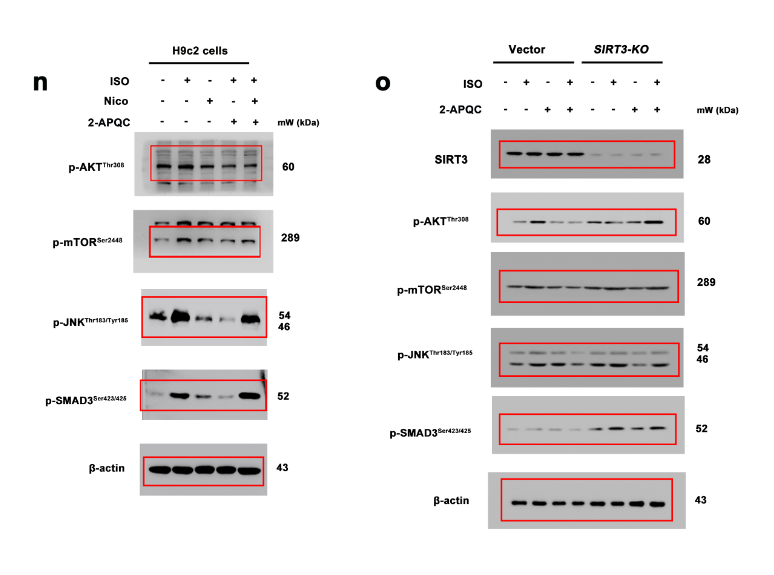
**

**
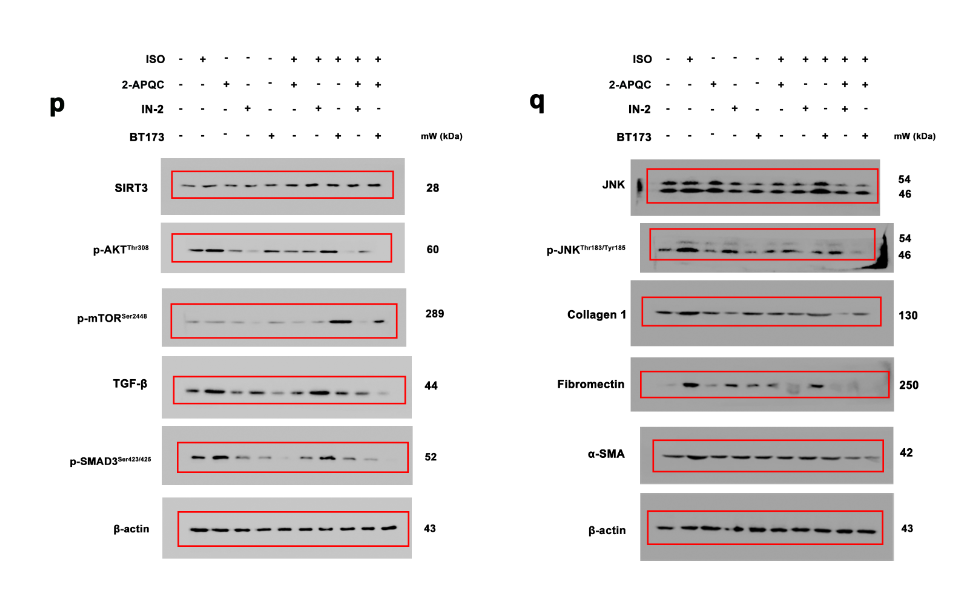
**

**Figure 4**

**
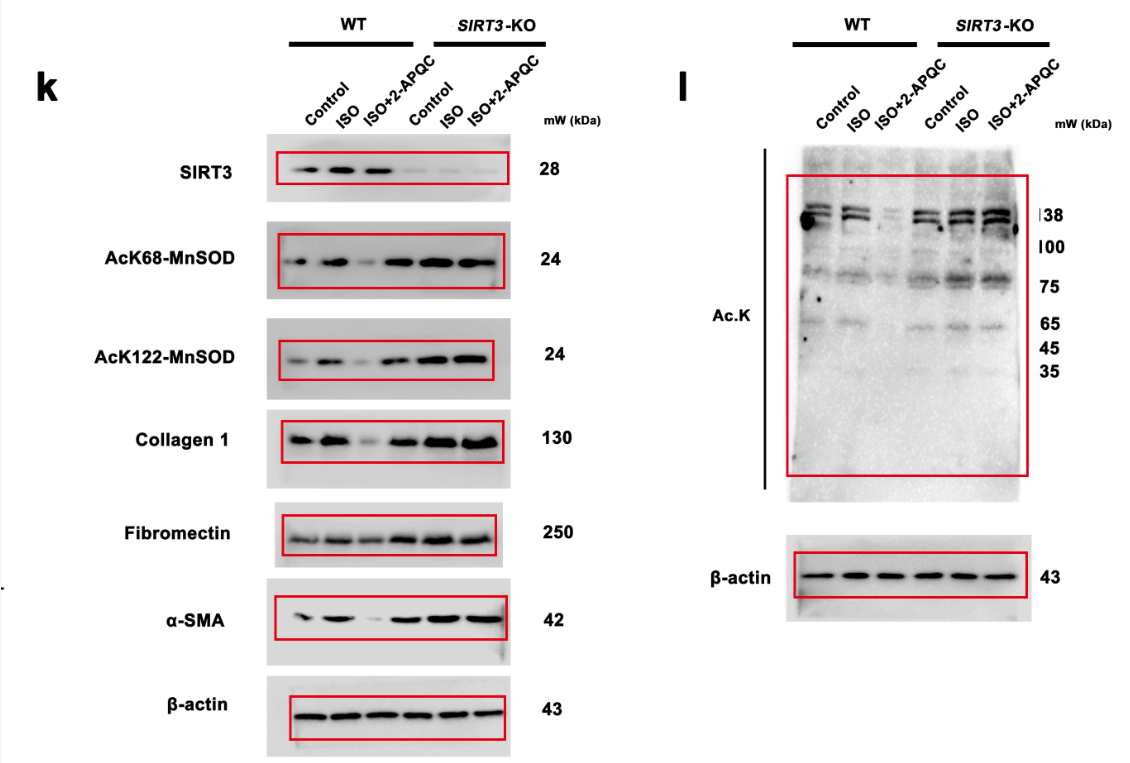
**

**Figure 6**

**
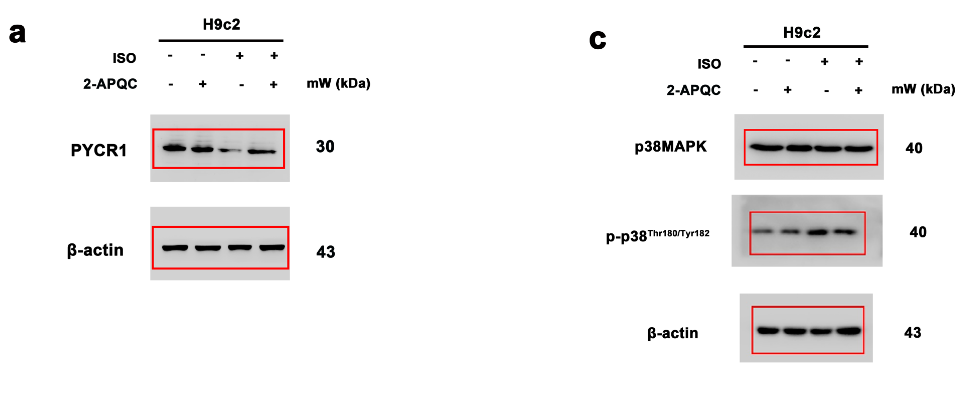
**

**
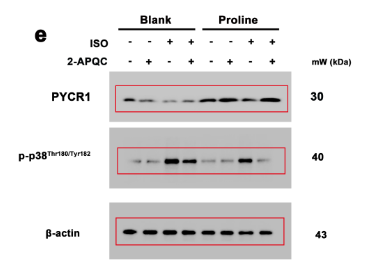
**

**
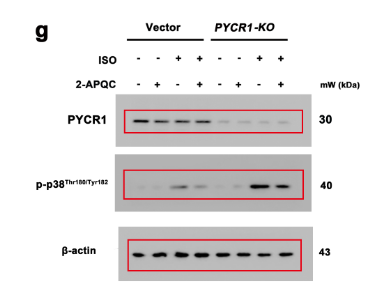
**

**
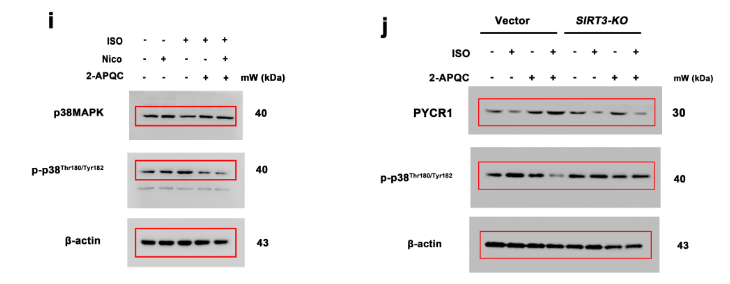
**

**
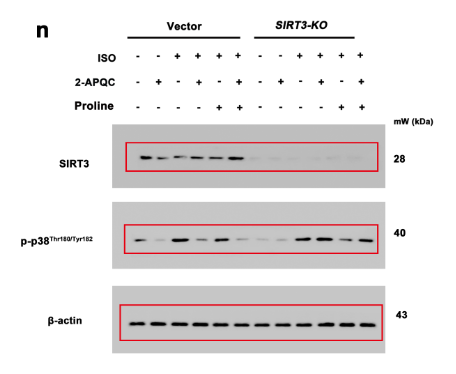
**

**
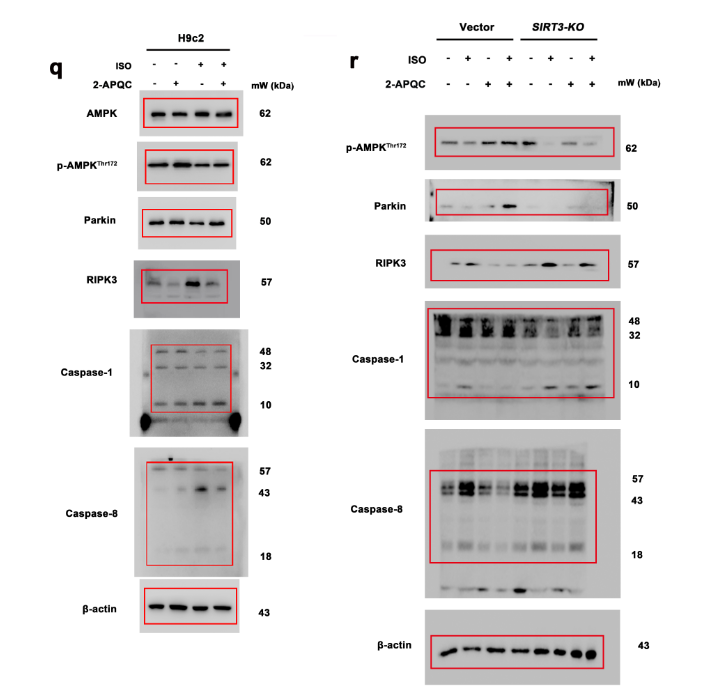
**

**Figure S3**

**

**

**

**

**
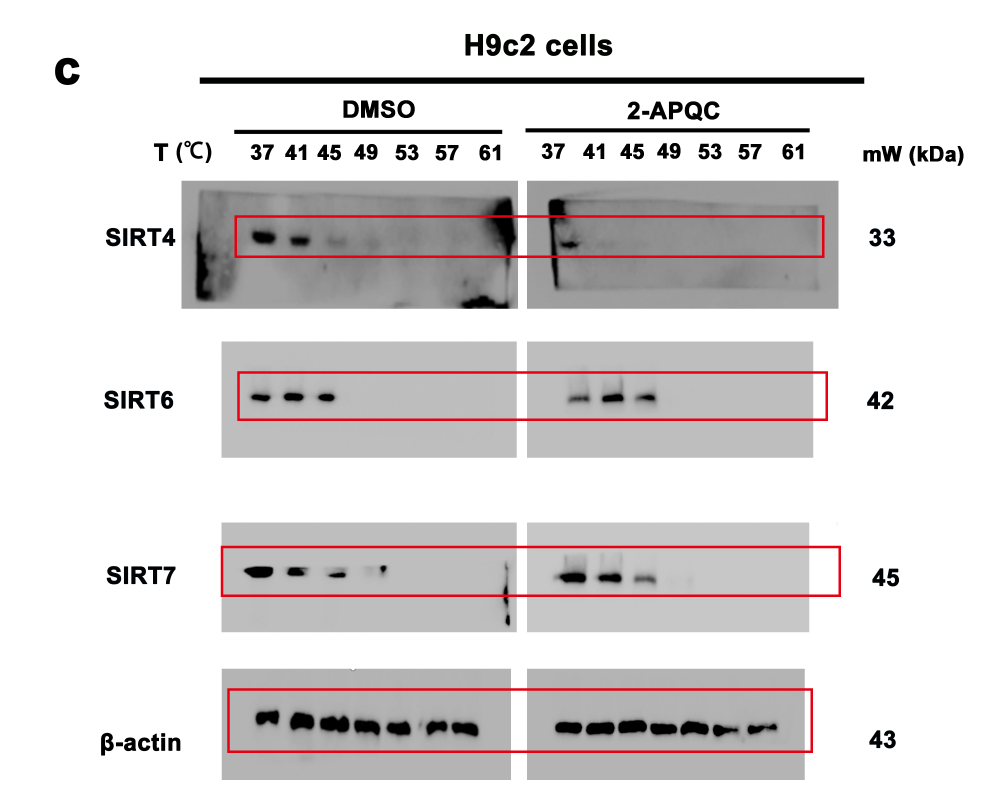
**

**

**
